# New Late Cretaceous titanosaur sauropod dinosaur egg clutches from lower Narmada valley, India: Palaeobiology and taphonomy

**DOI:** 10.1371/journal.pone.0278242

**Published:** 2023-01-18

**Authors:** Harsha Dhiman, Vishal Verma, Lourembam R. Singh, Vaibhav Miglani, Deepak Kumar Jha, Prasanta Sanyal, Sampat K. Tandon, Guntupalli V. R. Prasad

**Affiliations:** 1 Department of Geology, Center for Advanced Studies, University of Delhi, New Delhi, India; 2 Higher Secondary School, Bakaner, Dhar District, Madhya Pradesh, India; 3 G-46, Upper Ground Floor, Kirti Nagar, New Delhi, India; 4 Department of Earth Sciences, Indian Institute of Science Education and Research (IISER) Kolkata, Mohanpur, India; 5 Department of Earth and Environmental Sciences, IISER Bhopal, Bhopal, Madhya Pradesh, India; Indian Institute of Science, INDIA

## Abstract

The Upper Cretaceous (Maastrichtian) Lameta Formation is well-known for its osteological and oological remains of sauropods from the eastern and western parts of the Narmada Valley, central India. The newly documented ninety-two titanosaur clutches from Dhar District (Madhya Pradesh State, central India) add further to this extensive data. Previously parataxonomy of these titanosaur clutches was carried out with a few brief reports on palaeobiological and taphonomic aspects. The quantitative data collected from the new clutches (this study) opens avenues to additionally understand more about titanosaur palaeobiology and to qualitatively understand preservation and taphonomical aspects of their egg clutches. Herein, we document 256 eggs and three clutch patterns (viz. circular, combination, linear) that are assignable to six oospecies. The high oospecies diversity points to a possible high diversity in titanosaur taxa in the Indian sub-continent though it is not reflected in titanosaurid body fossils. All the macro- and micro-structures helped in understanding egg deformation and preservation from a taphonomic point of view. Additionally, a pathologic egg documented from the study area helped in understanding the reproductive biology of titanosaurs, such as the possibility of segmented oviduct and sequential laying of eggs by titanosaurs. In addition, we made an attempt to infer aspects such as egg burial, absence of parental care, colonial nesting behavior. All the egg clutches were observed within sandy limestone and calcareous sandstone lithologies that occur in scattered outcrops with rocks showing floating siliciclastic grains in a micritic groundmass. Further, the presence of ferruginous sandstone in the Jamniapura and Padlya regions (Dhar District, central India) is indicative of a possible alluvial/fluvial setting. The presence of grainy intraclastic fabric, alveolar-septal fabrics, brecciation and shrinkage cracks observed in the clutch-bearing rocks are indicative of a low energy-low gradient palustrine depositional condition in a fluvial/alluvial setting. Finally, we envisage that a few egg clutches of this area were laid close to lake/pond margins while most were laid away from the lake/pond margins, and thus, were hatched.

## Introduction

Peninsular India is well known for dinosaur oological fossils that occur at several sites in the Lameta Formation of central and western India, Deccan intertrappean beds, and shallow marine formations of the Cauvery Basin marking the extensive spread of the Indian dinosaur clutch, egg and eggshell sites [1–15]. From the Lameta Formation, dinosaur clutches, isolated eggs and eggshell fragments have been reported from the Lameta Ghat (type section), Bara Simla Hill, Chui Hill in Jabalpur, Bagh and Kukshi areas of Dhar District and Betul District of Madhya Pradesh (M.P.), Rahioli-Balasinor area of Gujarat, and Nand-Dongargaon Basin in Chandrapur District, Maharashtra.

Since the first report of dinosaur bones from the type locality near Jabalpur by Captain Sleeman in 1928 [16], the Upper Cretaceous Lameta Formation has remained as an important source for well preserved and taxonomically identifiable dinosaur fossils which include both osteological and oological remains. Five theropod (*Rajasaurus narmadensis*, *Rahiolisaurus gujaratensis*, *Indosuchus raptorius*, *Indosaurus matleyi*, and *Laevisuchus indicus*) and three sauropod (*Jainosaurus* cf. *septentrionalis*, *Isisaurus colberti*, and Titanosauriformes indet.) taxa [17] (see references therein) along with egg clutches, isolated eggs, and eggshell fragments belonging to nine oospecies of titanosaurs (*Megaloolithus cylindricus*, *M*. *jabalpurensis*, *M*. *megadermus*, *M*. *dhoridungriensis*, *M*. *khempurensis*, *Fusioolithus mohabeyi*, *F*. *baghensis*, *F*. *dholiyaensis*, and *F*. *padiyalensis*) [7, 9, 11] and probable theropods (*Subtiliolithus kachchhensis* and *Ellipsoolithus khedaensis*) [7, 9, 18] have been documented, to date.

**Fig 1:**
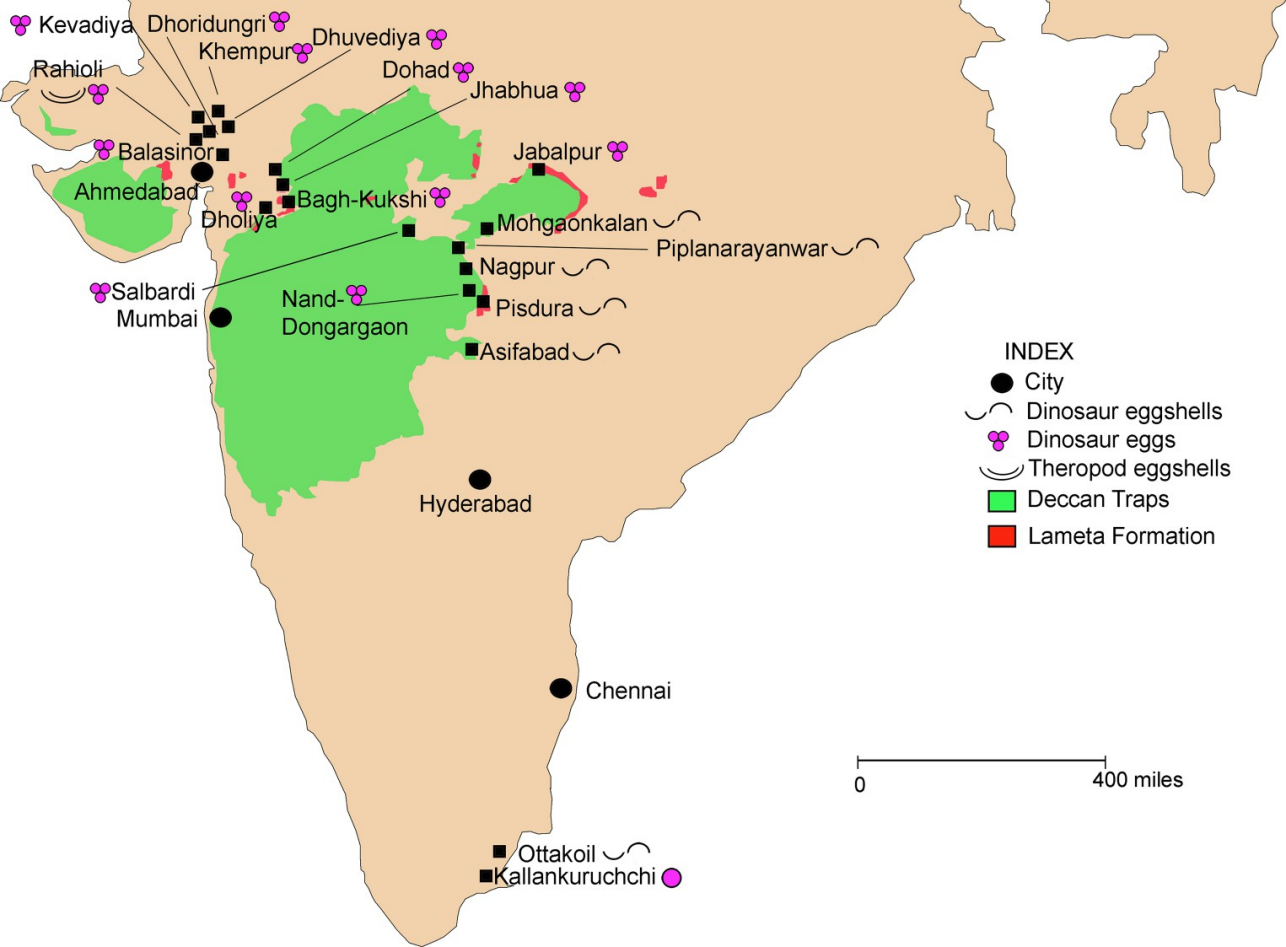
Map of India showing the distribution of Late Cretaceous dinosaur eggs and nesting sites (modified after Mankar and Srivastava [25]; outline of India—courtesy of the University of Texas Libraries, The University of Texas at Austin).

Of all the above mentioned sites, it is only in the type locality at Jabalpur that an attempt was made to understand taphonomy, palaeobiology, and palaeoenvironment of the dinosaur clutches [6]. In other sites, such as Balasinor in Kheda District in Gujarat, Salbardi in Betul District, M.P. and Pisdura-Nand-Dongargaon Basin in Maharashtra (Fig 1) only parataxonomic classification of eggs and eggshells was carried out [3, 4, 7, 9–13, 18]. Limited stable isotope (Carbon and Oxygen) analyses of eggshells and host pedogenic carbonates from Balasinor and Jabalpur areas have also been carried out [19–22]. Comparative studies on titanosaur nesting sites from several localities of Argentina, Spain, France, and India (Lameta Formation) and the reproductive biology of sauropod dinosaurs have also been undertaken [23, 24]. However, no integrated study incorporating work on the preservational conditions, depositional environments, palaeobiology, and palaeoclimate of the Lameta nesting sites of the lower Narmada valley has been carried out, to date.

**Fig 2:**
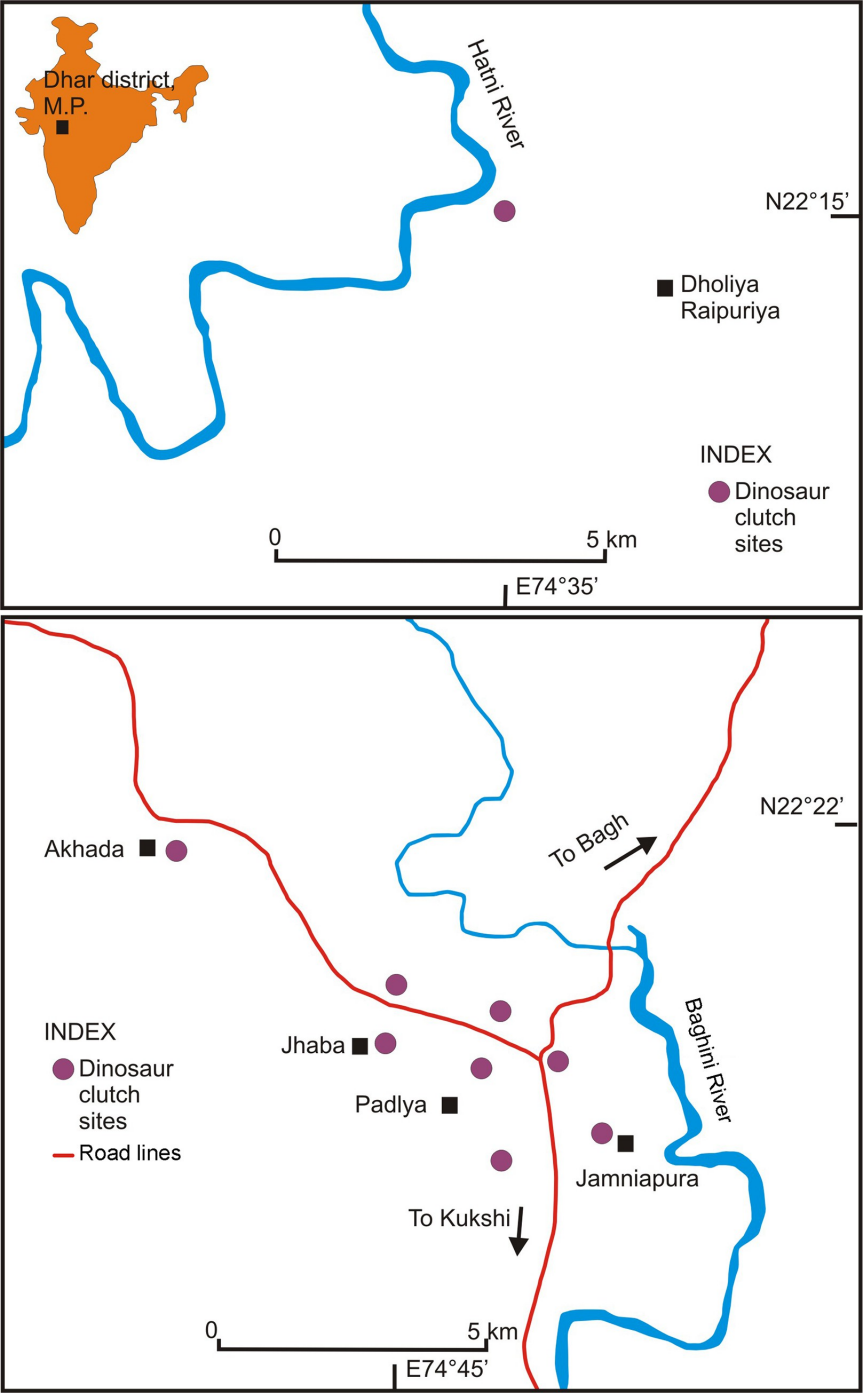
Map of the study area displaying the location of investigated dinosaur clutches (courtesy of the University of Texas Libraries, The University of Texas at Austin).

During the field investigations carried out between 2017 and 2020, we found extensive hatcheries of dinosaurs in Bagh and Kukshi areas in Dhar District, M.P., notably from the villages Akhada, Dholiya Raipuriya, Jhaba, Jamniapura, and Padlya (Fig 2). This region falls in between the eastern most Lameta exposures at Jabalpur in upper Narmada valley (central India) and Balasinor in the west in lower Narmada valley (western central India). The Bagh-Kukshi area with many new nesting sites (n = 92) that have greater areal spread offer a fair amount of qualitative and quantitative nesting data. Currently, there exists a knowledge gap in our understanding of sauropod dinosaur nesting patterns, their preservational conditions and depositional environments, and palaeobiology. Thus, we herein present an integrated study incorporating parataxonomy, nesting behaviour, reproductive biology, sedimentology, taphonomy, organic [15] and stable isotope geochemistry (S1 File), and palaeobiogeography [14] of the newly discovered clutches in Bagh-Kukshi area and discuss our observations related to the palaeobiology and taphonomy of the Indian Late Cretaceous titanosaurs.

## Geological description of the Lameta Formation

Pericratonic basins formed along the eastern and western margins of India during the Mesozoic Era due to several reactivation episodes related to the break-up of the Pangean Supercontinent [26]. These basins have been subjected to phases of transgressions from the west through the Narmada valley and also from the southeastern coast of India through the Godavari valley [26–29]. In the Narmada valley, these marine incursions resulted in the deposition of the Upper Cretaceous (Cenomanian to Coniacian) Bagh Group of rocks [30–32]. Following the withdrawal of marine seaway from the Narmada valley during the Late Cretaceous (Coniacian), the deposition of arenaceous, argillaceous, and calcareous sediments commenced in continental palaeoenvironments [33]. These strata designated as the ‘Lameta Formation’ represent a lithostratigraphic unit that generally occurs below some of the oldest basaltic flows of the Deccan Traps and, in places may also occur interbedded with them [34].

The Upper Cretaceous (Maastrichtian) Lameta Formation has a wide geographic distribution in central (Madhya Pradesh, Maharashtra) and western India (Gujarat). The Lameta Formation is well-known to yield skeletal remains of fishes, turtles, snakes, and dinosaurs, coprolites, and plant remains in the form of petrified wood and palynoflora [17] (see references therein). On the basis of faunal and palynofossil evidences, and magnetostratigraphy, it has been assigned a late Cretaceous (Maastrichtian) age [35–39]. There are two diverging views on the depositional environment of the Lameta Formation. Classically, on the basis of purported occurrence of glauconite in the basal Green Sandstone, supposed *Thalassinoides* burrows in the Mottled Nodular Bed, and algal structures, a shallow marine environment has been suggested [40–44]. Alternatively, a continental environment in a fluvio-lacustrine setting was favoured based on terrestrial fossils, structures related to pedogenesis in a semi-arid environmental setting, and carbonate-chert of fluvio-limnic associations [21, 45–50]. Here we subscribe to the latter view of fluvio-lacustrine environment of deposition in a semi-arid climate for the Lameta Formation of the studied area as we did not observe any sedimentological and fossil evidences for a marine depositional environment.

**Fig 3:**
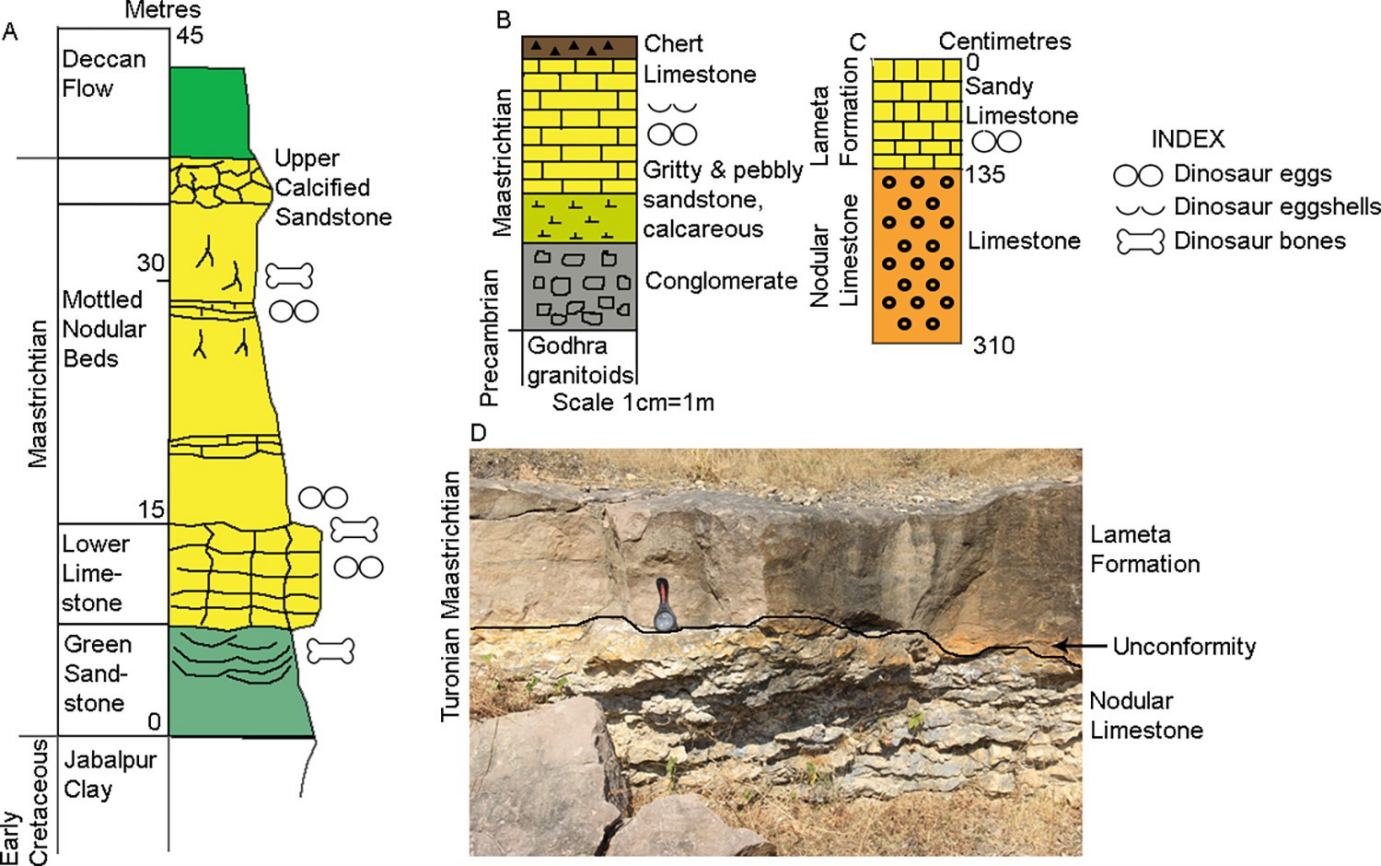
Stratigraphic subdivisions of the Lameta Formation in the type section at Jabalpur (A) (modified after Tandon et al. [21]), Rahioli (B) (modified after Srivastava et al. [4]), and Padlya, Dhar District, M.P. (C) (modified after Dhiman et al. [51]). (D) Field photograph showing the Sandy Limestone of the Lameta Formation overlying the Nodular Limestone of the Bagh Group within Dinosaur Fossil National Park (DFNP), near Padlya, Dhar District, M.P. The Nodular Limestone shows lensoid character while the sandy limestone has massive appearance.

In the type section near Jabalpur in the upper Narmada valley, the Lameta Formation is divided into the basal Green Sandstone, Lower Limestone, Mottled Nodular Bed, and Upper Calcareous Sandstone in this order of superposition [21] (Fig 3). The dinosaur clutches both at Jabalpur and Balasinor occur in the Lower Limestone and, rarely in the Mottled Nodular Beds [4, 21]. However, such stratigraphical divisions are not discernible in the study areas of Bagh-Kukshi region of Dhar District (Fig 3). The dinosaur clutches from the study area were retrieved from sandy limestone and calcareous sandstone lithologies of the Lameta Formation which are similar in lithology to that of the previously documented dinosaur clutches from Jabalpur and Balasinor (the Lower Limestone horizon). We consider the dinosaur clutch-bearing horizon in our study areas near Padlya and Dholiya Raipuriya villages in the Dhar District of Madhya Pradesh, lying in between Jabalpur and Balasinor, as representing a facies similar to the Lower Limestone of the type section.

## Material and methods

### Field work

Detailed field investigations were conducted in December 2017, January 2018 and March 2020 in the Dhar District, Madhya Pradesh, central India. The data with regards to nest type, number of eggs, diameter of eggs, egg-shape, egg-type, and pathology were collected as field observations related to egg clutches (Table 1). Additionally, along with the fossilized eggshells, specimens of host rocks of some of the egg clutches were also collected and data on their sedimentological characteristics was also recorded.

**Table 1:** Diagnostic characters of the titanosaurid dinosaur clutches from the study areas of the Lameta Formation from Dhar (M.P.) District, India (abbreviations stand for A-Akhada, DR-Dholiya Raipuriya, J-Jhaba, JMP-Jamniapura, P-Padlya).

Nest#	Nest type/Material	No. of eggs	Min to max diameter of egg outline/egg (in cm)	Shape of egg outline/egg	Eggshells	Hatching windows	Shell pile/Double bottom	Egg type
A1	Eggshells	1	-	-	Random	-	-	-
A2	Eggshells	1	-	-	Random	-	-	-
A3	Eggshells	1	-	-	Random	-	-	-
A4	Eggshells	1	-	-	Random	-	-	-
A5	Eggshells	1	-	-	Random	-	-	-
DR1	Eggshells	1	-	-	Random	-	-	-
DR2	Linear	3	7–15	Elliptical	Outside (concentric) and inside egg outline	-	Egg vi	Fragmented and compressed (vi)
DR3	Eggshells	1	-	-	Closely spaced and concentric	-	-	-
DR4	Circular	3	6–10	Elliptical	Random and inside	-	Egg i	Compressed (i); Bottom (ii, iv)
DR5	Linear	1	-	-	Linear and concentric	-	-	-
DR6	Linear	2	-	-	Linear	-	-	-
DR7	Linear	1	-	-	Linear and concentric	-	-	-
DR8	Linear	4	5–17	Elliptical	Linear and concentric	-	-	Compressed and fragmented (i)
DR9	Combination	5	6.1–15	Sub-circular	Inside	Egg i	Egg v	Fragmented (i-v)
DR10	Two eggs	2	3.9–16.8	Sub-circular to elliptical	Outside	Egg ii	-	Fragmented (i); Compressed (ii)
DR11	Linear	2	-	-	Linear and concentric	-	-	-
DR12	Eggshells	1	-	-	Random	-	-	-
DR13	Two eggs	2	3–15.8	Sub-circular	Outside	-		Fragmented (i,ii)
DR14	Eggshells	1	-	-	Random	-	-	-
DR15	Linear	1	-	-	Linear and concentric	-	-	-
DR16	Linear	1	-	-	Linear and concentric	-	-	-
J1	Two eggs	2	9.3–15.3	Sub-circular	Outside	-	-	Partially intact and morphed (i,ii)
J2	Two eggs	2	5.5–14	Elliptical	Inside and outside	-	Egg iii	Fragmented and compressed (i); Half-preserved (B)
J3	Single egg	1	16.2–16.5	Circular	-	-	-	Fragmented
J4	Circular	4	10–14.8	Sub-circular	Inside	-	-	Fragmented (i-iv)
J5	Two eggs	2	9.3–15.5	Sub-circular	Inside and outside	-	-	Fragmented (i,ii)
J6	Single egg	1	13.2–13.5	Circular	-	-	-	Partially intact and eroded
J7	Circular	4	11.3–15.3	Elliptical	Inside	Egg i	Egg i	Compressed (i); Remnants (ii-iv)
J8	Two eggs	2	8.1–17	Elliptical	Inside	Egg ii	Egg ii	Fragmented and compressed (i,ii)
J9	Single egg	1	-	-	Outside	-	-	Bottom
J10	Single egg	1	11.1–12.7	Sub-circular	Inside	Present	-	Fragmented
J11	Circular	3	-	-	Inside	Eggs i,ii	Eggs i,ii	Half-preserved (i,ii); Compressed (ii); Remnant (iii)
J12	Eggshells	1	-	-	Closely spaced	-	-	-
J13	Single egg	1	17.6–19	Sub-circular	-	-	-	Unhatched
J14	Two eggs	2	-	Sub-circular	-	-	-	Bottom (i,ii)
J15	Single egg	1	15–15.4	Circular	-	-	-	Half-preserved
J16	Single egg	1	-	-	-	-	-	Fragmented
J17	Single egg	1	-	-	-	-	-	Stolen
J18	Circular	5	-	-	-	-	-	Bottom; Other eggs stolen
JMP1	Double eggs	2	18	Sub-circular	Outside	-	-	Fragmented
P1	Combination	20	9.8–18.8	Sub-circular	-	Eggs ii,vi,vii,xiii-xvii	-	Intact (i,iii,viii,x,xi); Partially intact and half-preserved (ii,vi,vii,xiii); Bottom (iv,v,xii); Compressed (xi); Fragmented (xiv-xx)
P2	Circular	3	15.5–16.6	Sub-circular	-	Egg i	-	Fragmented (i,ii); Remnant (iii)
P3	Two eggs	2	12.5–15.5	Sub-circular	-	-	-	Fragmented (i,ii)
P4	Single egg	1	-	-	-	-	-	Bottom
P5	Single egg	1	14.7–15.5	Circular	-	-	-	Intact but eroded
P6	Single egg	1	16.1–16.8	Sub-circular	-	-	-	Partially intact
P7	Circular	10	13–16.6	Sub-circular to elliptical	Inside and outside	Eggs i,ii	Egg xiv	Half-preserved (i,ii); Unhatched (iii); Bottom (iv,vii); Intact and eroded (viii,ix); Remnant (L,M); Compressed (N)
P8	Circular	5	15–19	Sub-circular	-	-	-	Partially intact (i,iii); Bowl-shaped (ii); Half-preserved (v); Bottom (iv)
P9	Circular	6	13.2–18.3	Sub-circular to elliptical	-	-	-	Fragmented (i,ii,iv,v); Remnant (iii); Compressed (vi)
P10	Two eggs	2	-	-	-	-	-	Bottom (i,ii)
P11	Circular	5	13.4–15.8	Sub-circular	-	-	-	Bowl-shaped (i); Bottom (ii-v)
P12	Circular	4	13.1–19.6	Sub-circular	-	-	-	Bottom (i,iv); Half-preserved (ii,iii)
P13	Two eggs	2	15–16.3	Circular	Inside	-	-	Partially intact and eroded (i); Remnant (ii)
P14	Combination	4	12.7–18.3	Sub-circular	Inside and outside	Eggs i-iv	Egg iv	Half-preserved and fragmented (i-iv)
P15	Circular	8	7.2–15.5	Circular to sub-circular	Inside and outside	-	-	Partially intact (ii,iii,vii); Compressed (v); Bowl-shaped (vi); Half-preserved (viii)
P16	Circular	4	12–13.6	Sub-circular	-	-	-	Fragmented (i); Bowl-shaped (iv)
P17	Two eggs	2	16.8–17.6	Sub-circular	Inside	Egg ii	Egg ii	Fragmented and half-preserved (i,ii)
P18	Single egg	1	15.3–16	Sub-circular	Outside	-	-	Partially intact and displaced
P19	Circular	3	10.5–13.5	Sub-circular	Inside	Egg i	Egg i	Half-preserved (i,iii); Remnant (ii)
P20	Circular	8	9.5–14.5	Sub-circular	Inside and outside	Eggs v,vi	Eggs v,vi	Unhatched (i,ii); Remnant (iii,vii); Fragmented (iv); Half-preserved (v,vi)
P21	Circular	4	-	Sub-circular	-	-	-	Bottom (i-iv)
P22	Circular	14	4.5–15.8	Sub-circular	Inside and outside	Eggs i,iv	Egg x	Half-preserved (i,iv,x); Bottom (ii); Fragmented (ix); Compressed (xi); Remnant (xii); Partially intact (xiv)
P23	Two eggs	2	8.2–13.5	Sub-circular	Inside and outside	Egg iii	Egg iii	Fragmented (ii,iii)
P24	Circular	4	9–10	Sub-circular	Outside	-	-	Fragmented (ii); Remnants (iii,iv)
P25	Circular	3	15–18.8	Sub-circular to elliptical	Inside	Eggs i-iii	Eggs i,iii	Fragmented (i-iii)
P26	Circular	8	8.5–16.1	Sub-circular to elliptical	Inside and outside	-	-	Fragmented (i,iii,x); Bottom (ii); Compressed (xi)
P27	Eggshells	1	-	-	Random	-	-	-
P28	Single egg	1	13.1	-	Inside	Present	Present	Half-preserved
P29	Single egg	1	17.7–19.3	Sub-circular	-	-	-	Unhatched
P30	Single egg	1	16.7–20.3	Elliptical	-	-	-	Unhatched and deformed
P31	Single egg	1	8.5–17.5	Elliptical	Inside	Present	Present	Half-preserved
P32	Single egg	1	14.2–17.2	Elliptical	-	-	-	Unhatched
P33	Two eggs	2	13.3–16.4	Circular	-	-	-	Intact and eroded (i,ii)
P34	Two eggs	2	5.8–15	Sub-circular	-	-	-	Bottom (i,ii)
P35	Circular	6	8.3–16.3	Circular to sub-circular	-	-	-	Intact (i,ii,iii,v); Bottom (iv); Small sized (vi)
P36	Eggshells	1	-	-	Random	-	-	-
P37	Single egg	1	16.3–18.6	Sub-circular	-	-	-	Unhatched
P38	Two eggs	2	10.2	-	Inside	Egg i	Egg i	Half-preserved (i,ii)
P39	Single egg	1	-	-	-	-	-	Bottom
P40	Two eggs	2	-	-	-	-	-	Bottom (i,ii)
P41	Single egg	1	-	-	-	-	-	Bottom
P42	Eggshells	1	-	-	Random	-	-	-
P43	Single egg	1	16.9–17	Circular	-	-	-	Unhatched
P44	Two eggs	2	11.5–12.1	-	-	-	-	Half-preserved (i,ii)
P45	Eggshell	1	-	-	Single	-	-	-
P46	Two eggs	2	14.2	-	-	-	-	Bowl-shaped (i)
P47	Circular	5	-	-	-	-	-	Bottom (i-v)
P48	Two eggs	2	-	-	Closely spaced	-	-	Bottom (i)
P49	Two eggs	2	-	-	-	-	-	Bottom (i,ii)
P50	Single egg	1	14	-	Inside	Present	Present	Half-preserved
P51	Circular	12	10.4–15.2	Sub-circular to circular	Outside	-	-	Bottom (i-iv,ix,xii); Remnants (v-xi); Half-bowl (viii)
P52	Circular	3	9–13.5	Sub-circular	-	-	-	Fragmented (i); Partially intact (ii,iii)

### Cataloguing and cleaning

The rock matrix was removed from the eggs and eggshell specimens using fine (0.2 to 1.0 mm) needles under Stereoscopic Binocular Zoom Microscope (Nikon SMZ 745) and labelled as per the number of clutch site and location in the Vertebrate Palaeontology Laboratory, Department of Geology, University of Delhi. This was followed by cleaning of the specimens with ethanol for a few seconds and then transferring to an ultrasonic bath for one to two minutes depending on the specimen size followed by air-drying.

### Photomicrography

Subsequently, photomicrography of individual eggshells to be used for thin section study was carried out using Leica S8 AP0 Stereoscopic Zoom microscope attached with Leica MC120HD digital camera in the Vertebrate Palaeontology Laboratory, Department of Geology, University of Delhi. Microstructural details of external, internal, and radial surfaces were recorded.

### Scanning electron microscope (SEM) photography

Few selected eggshell specimens were also pasted on 1 cm diameter aluminium stubs using carbon tape and coated with gold-palladium in vacuum for 10–15 minutes. They were then studied under JEOL Neoscope 6000 Plus Benchtop SEM with Energy Dispersive X-Ray Spectroscopy in the Metamorphic Petrology Lab, Department of Geology, University of Delhi to understand their ultrastructure which reveals the morphology of the shell units, pore canals, external nodes, and internal resorption craters through surface imaging.

### Histology

Separately, petrological thin sections of rocks and eggshells were prepared using standard procedure [52]. It is important to mention that rock and eggshell specimens were collected from numerous clutches barring a few clutches owing to lack of well preserved material and hostility from neighbouring villagers. The collected rock specimens were initially cleaved and soaked in resin for consolidation. However, this exercise was avoided in the case of eggshell specimens to avoid damage caused to specimens by the hot resin. The specimens were ground and polished using carborundum powder of various grades (400–1000 μm). After making the radial section of the eggshells flat, the flattened surfaces were pasted on glass slides using araldite and were left to dry for 12–24 hours. They were ground and polished to achieve a thickness of 0.03 mm and the final step in grinding was completed with diamond polishing. A total of forty-six eggshell thin sections were examined under the petrological microscope to study the eggshell morphology. Photomicrographs of the thin sections were taken using Carl Zeiss Axio Imager A1m High Resolution Petrological Microscope in the Vertebrate Palaeontology Laboratory and Nikon Eclipse 50i Polarizing Microscope with Nikon’s Digital Sight DS-U3 camera attachment in Metamorphic Petrology Laboratory of the Department of Geology, University of Delhi.

### X-Ray Microscopy (XRM)

XRM was performed on two dinosaur eggshell fragments (specimen numbers P7 and P11) at the Indian Institute of Technology (Bombay) using Zeiss Xradia 520 Versa 3D X-Ray Microscope to have cross-sectional views of the eggshells. Using XRM, 3D photographs along with cross-sectional 2D slices of the specimens were obtained. Further 3D digital volumes of the specimens were produced after importing the data sets into ORS visual SI software. The 2D images were stacked to recreate 3D volumes.

### Repository

The specimen names are abbreviated after the field location (for example Akhada–A; Dholiya Raipuriya–DR; Jamniapura–JMP; Jhaba–J; Padlya–P) and then numbered according to the clutch numbers recorded during the field work. The specimens are housed at Vertebrate Palaeontology Lab, Department of Geology, University of Delhi, except specimen number J13, P29, P30, P32, P37, and P43 (complete eggs) which are housed in Haripad Anand Math, private museum collections at Manawar, Dhar District, M.P. Some of the nests (clutches P2 to P15, P20, P21) of village Padlya are now relocated to Dinosaur Fossil National Park (DFNP), Bagh and Ashamadha Fossilarium at Mandu, Dhar District, M.P. No permits were required for the described study, which complied with all relevant regulations.

## Field observations

### Distribution of dinosaur clutches

**Fig 4:**
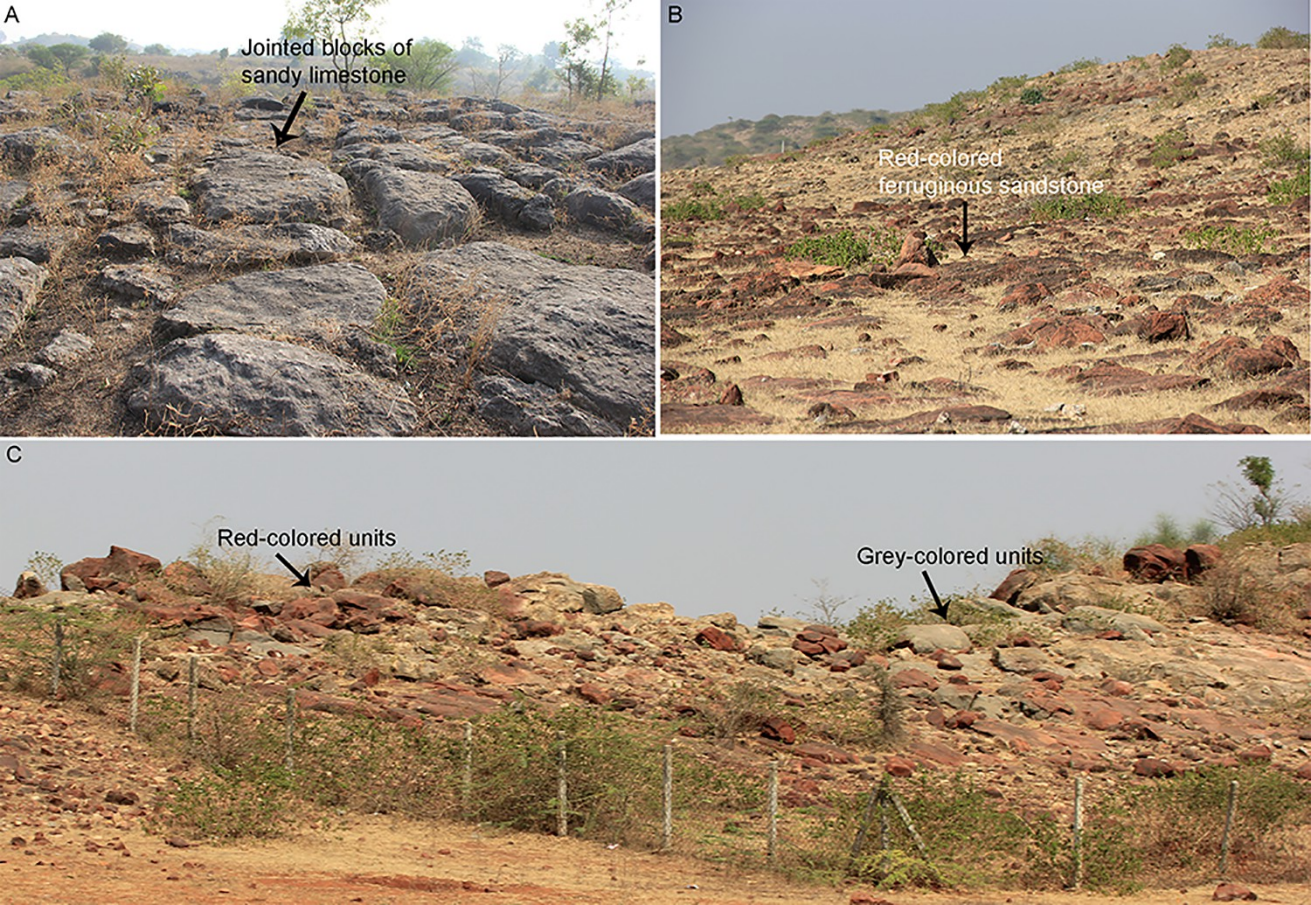
Field photographs showing outcrop characteristics. (A) Outcrop near Dholiya Raipuriya, M.P. showing variation in the calcareous content expressed in terms of the patchy colour changes; also note the blocky character of these sandy limestone outcrops because of the joints. (B) Outcrops of red-coloured ferruginous sandstones at Padlya, M.P.; also note the variation in colour most likely due to differences in iron oxide content. (C) Red and grey-coloured rocks occurring in close association with each other within DFNP at Padlya, Dhar District, M.P. Note the prominent blocky nature of the outcrops.

Field data was collected from five localities, namely, Akhada, Dholiya Raipuriya, Jhaba, Jamniapura, and Padlya of Bagh-Kukshi areas (Fig 1). The Lameta beds in these localities occur as scattered and laterally extensive outcrops of sandy carbonate and calcareous sandstone lithologies (Fig 4) in which the titanosaur sauropod eggs are documented either as clutches or in the form of broken eggs with eggshell fragments scattered around. Since rocks from the studied sites exhibit lithologies that range from sandy limestone to calcareous sandstone, on the basis of the content of microcrystalline calcite, here we use the term sandy limestone for the clutch-bearing lithounit in order to avoid any confusion. This grey-coloured sandy carbonate outcrop also changes to maroon colour at some places. A third lithology of red-coloured ferruginous sandstone outcrops also occurs but only close to Jamniapura and Padlya villages and do not preserve dinosaur eggs (Fig 4).

A total number of 92 clutches have been documented from the five villages mentioned above. Near Akhada village, five scattered outcrops were observed (A1-A5) within an area of 183 m^2^. The oospecies documented from the Akhada village are *M*. *cylindricus* [7], *F*. *baghensis* [7, 11], and *F*. *padiyalensis* [7, 11]. Unfortunately, no data on individual eggs (in terms of diameter, number of eggs) could be recorded at Akhada village as only randomly scattered eggshell fragments were observed in the outcrops at this locality. Further, as mentioned earlier, no physical parameter data could be collected at this locality owing to hostility from the villagers.

A total number of 16 clutches (DR1-DR16) have been documented from an area of 4335 m^2^ from Dholiya Raipuriya village. The oospecies documented are *M*. *cylindricus*, *M*. *jabalpurensis* [7], *F*. *mohabeyi* [7, 11], and *F*. *padiyalensis*. The oological material is documented in the form of random, closely spaced, linear, and concentric eggshells, fragmented and compressed egg outlines, bottom surfaces of eggs, possible hatching windows and double bottom. The clutch type consists of circular, combination, and linear pattern. The titanosaur eggs are seen as circular to elliptical shaped egg outlines (sections) in two-dimensions. It is important to keep in mind that the diameter values represent the values measured directly from such egg outlines where larger value is measured right in the equatorial section and lower value comes from diametrically opposite position. On this basis the minimum and maximum diameters shown by egg outlines are 3 cm and 17 cm, respectively. The total number of complete eggs documented is 10. However, if eggshell aggregates are also taken into account then it’s safe to assume that the eggshell aggregates represent at least a minimum of one egg. On the basis of this assumption, 31 eggs are counted from a total of 16 clutches.

From Jhaba village, 18 clutches have been documented (J1-J18) within an area of 293,428 m^2^. The oospecies documented are *M*. *cylindricus*, *M*. *jabalpurensis*, *M*. *dhoridungriensis* [9], *F*. *baghensis*, and *F*. *padiyalensis*. The oological material is in the form of closely spaced eggshells, partially intact, fragmented, compressed and morphed egg outlines, bottom surfaces and unhatched eggs, hatching windows, shell fragment pile, double bottom and remnants of egg material. The clutch type documented is circular. The minimum and maximum diameters shown by egg outlines are 5.5 cm and 19 cm, respectively. The number of eggs is 35. The diametrical values shown by a partially spherical egg is between 15 cm and 15.4 cm. Another unhatched but slightly compressed egg shows diameter between 17.6 cm and 19 cm.

Several closely spaced blocks of maroon-coloured rocks exhibiting black-coloured eggshells are documented from a site near Jamniapura (JMP1). The oospecies identified at this locality is *F*. *baghensis*. Eggshell fragments and fragmented outlines were observed at this locality (Table 1). The estimated diameter of fragmented outlines is 18 cm and evidence for the presence of two eggs is documented.

Fifty two clutches (P1-P52) were identified from an area of 696,472 m^2^ from Padlya village. The oospecies recorded at this locality are *M*. *cylindricus*, *M*. *jabalpurensis*, *M*. *dhoridungriensis*, *F*. *mohabeyi*, and *F*. *baghensis*. The oological material is in the form of random, closely spaced, and concentric eggshells, fragmented, partially intact, and compressed egg outlines, bottom surfaces, bowl-shaped and unhatched eggs, hatching windows, double bottom, shell fragment pile, and remnants of egg material. The clutch types are circular and combination types. Minimum and maximum diameters of egg outlines are 4.5 cm and 20.3 cm, respectively and a total number of 183 eggs were recorded from this site (Table 1). Multi-shelled and ovum-in-ovo pathologies have been identified at this site. The diametrical values shown by slightly compressed eggs range from 14.2 cm to 20.3 cm while an isolated almost spherical egg shows values from 16.9 cm to 17 cm. Diagnostic characters of the clutches from the study areas are presented in Table 1.

Oospecies identification (parataxonomic classification)

For the purpose of parataxonomy, thin section studies were carried out on a total of sixty-nine eggshell specimens. Some of the eggshells showed strong effects of diagenetic alteration in the form of silicification affecting the morphology of the shell units in part or full and orientation of the growth lines. Thus, some of the diagenetically altered eggshells could not be utilized in terms of parataxonomy. Forty-six eggshells were finally selected and identified either definitely or tentatively on the basis of combined information from thin sections, stereo photomicrography, X-Ray Microscopy and SEM images. The diagnostic characters are listed in Table 2.

**Table 2:** Eggshell dimensions of the oospecies recorded from the study areas.

Nest#	Thickness (in mm)	Height/Width (H/W)	Average node diameter (in mm)	Average basal cap diameter (in mm)	Oospecies
A1	1.0–1.2	2.8:1	0.5	0.4	*Fusioolithus baghensis*
A3	2.2–2.7	4.1:1	0.6	0.3	*Megaloolithus cylindricus*
A4	1.4–1.7	2.4:1	0.6	0.4	*Fusioolithus baghensis*
A5	1.8–2.5	2.7:1	0.7	0.5	*Fusioolithus padiyalensis*
DR1	2–2.2	2.9:1	0.7	0.3	*Megaloolithus jabalpurensis*
DR6	2–2.1	5.2:1	0.6	0.2	*Megaloolithus cylindricus*
DR8	0.7–1.9	3.8:1	0.6	0.3	*Megaloolithus jabalpurensis*
DR9	1.2–1.5	3.7:1	0.4	0.2	*Fusioolithus mohabeyi*
DR10	1.7–2.1	3.5:1	0.6	0.4	*Fusioolithus padiyalensis*
DR11	1.5–2	2.8:1	0.6	0.6	*Megaloolithus jabalpurensis*
DR13	0.8–1.1	1.5:1	0.8	0.6	*Megaloolithus cylindricus*
DR14	1.1–1.4	3.5:1	0.3	0.2	*Megaloolithus cylindricus*
J9	1.1–1.8	3.6:1	0.4	0.3	*Fusioolithus baghensis*
J10	0.8–1.2	2:1	0.5	0.2	*Fusioolithus baghensis*
J11	0.4–1.1	2.7:1	0.5	0.2	*Fusioolithus baghensis*
J12	2.1–2.3	2.8:1	1.0	0.2	*Fusioolithus baghensis*
J13	2.1–3.2	4.5:1	0.7	0.3	*Megaloolithus cylindricus*
J14	1.4–1.9	3.1:1	0.8	0.3	*Megaloolithus dhoridungriensis*
J15	2.1–2.8	4.6:1	0.7	0.3	*Fusioolithus padiyalensis*
J17	1–1.3	1.4:1	0.6	0.5	*Megaloolithus jabalpurensis*
JMP1	1.4–2	1.4:1	0.9	0.3	*Fusioolithus baghensis*
P3	1.5–1.7	3.4:1	0.9	0.4	*Megaloolithus cylindricus*
P7	1.3–1.6	2.2:1	0.6	0.4	*Megaloolithus jabalpurensis*
P8	0.9–1.5	3:1	0.6	0.2	*Megaloolithus jabalpurensis*
P10	1.5–1.7	2.5:1	0.6	0.2	*Megaloolithus jabalpurensis*
P11	1.4–1.6	2.6:1	0.6	0.4	*Megaloolithus jabalpurensis*
P12	1.2–1.5	2.1:1	0.8	0.2	*Megaloolithus dhoridungriensis*
P16	0.6–0.9	1.5:1	0.3	0.1	*Megaloolithus jabalpurensis*
P19	1.6–1.8	2.6:1	0.6	0.4	*Megaloolithus cylindricus*
P22	1.3–1.8	2:1	0.8	0.5	*Fusioolithus mohabeyi*
P23	0.4–0.9	1.2:1	0.7	0.2	*Fusioolithus baghensis*
P25	1.2–1.3	2.1:1	0.6	0.4	*Fusioolithus baghensis*
P27	1–1.5	2.5:1	0.5	0.2	*Fusioolithus mohabeyi*
P28	1.9–2	2.8:1	0.5	0.3	*Megaloolithus cylindricus*
P29	1.6–2.1	5.2:1	0.4	0.2	*Megaloolithus cylindricus*
P30	1.3–1.5	2.1:1	0.5	0.3	*Megaloolithus cylindricus*
P32	1.8–2.1	4.2:1	0.6	0.3	*Megaloolithus cylindricus*
P37	1.6–1.9	2.2:1	0.8	0.5	*Megaloolithus jabalpurensis*
P39	0.7–1.5	2.1:1	0.7	0.3	*Megaloolithus jabalpurensis*
P40	0.8–1.1	2.7:1	0.5	0.4	*Fusioolithus baghensis*
P42	1.5–2.3	5.7:1	0.6	0.2	*Megaloolithus cylindricus*
P44	0.9–1.8	3.6:1	0.6	0.3	*Fusioolithus mohabeyi*
P47	1.9–2.2	3.6:1	0.7	0.2	*Megaloolithus dhoridungriensis*
P49	1.6–1.8	2.5:1	0.5	0.3	*Megaloolithus cylindricus*
P51	1.7–2.1	3.5:1	0.6	0.3	*Megaloolithus cylindricus*
P52	1–1.2	2:1	0.6	0.1	*Fusioolithus baghensis*

**Fig 5:**
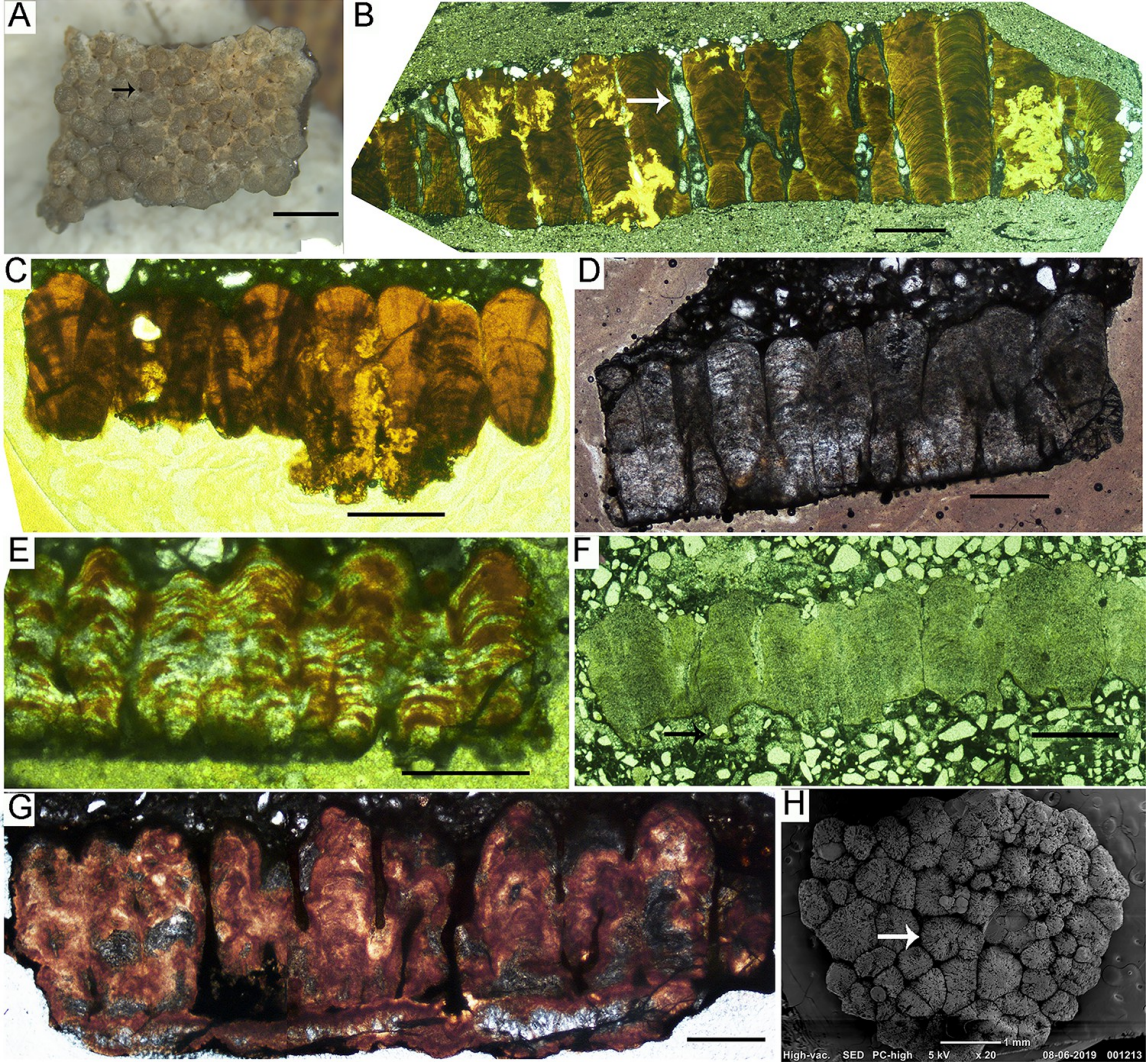
Microstructural details of the eggshells. (A) Stereoscopic binocular microscope photomicrograph of the tangential image of eggshell from clutch P32 showing compactituberculate ornamentation in the form of dense nodes separated by pore spaces (see arrow) (Scale bar: 2 mm). (B) Radial thin section of eggshell oospecies *Megaloolithus cylindricus* from clutch A3 showing long and cylindrical shell units with few areas of diagenetic alteration and vertical tubocanaliculate pore canals (see white arrow). The growth lines are limited to individual shell units (after Dhiman et al. [15]). (C) Radial thin section of *M*. *jabalpurensis* eggshell from clutch P11 showing fan-shaped shell units and arching growth lines. (D) Highly altered radial thin section of the oospecies *M*. *dhoridungriensis* from clutch P47 showing conical shell units which are broader in the upper part. (E) Radial thin section of eggshell representing oospecies *F*. *mohabeyi* from clutch P22 showing fused shell units and growth lines merging with each other (after Dhiman et al. [15]). (F) Radial thin section of *F*. *baghensis* eggshells from clutch J9 showing fused shell units and swollen basal end units. (G) Radial thin section of *F*. *padiyalensis* eggshells from clutch A5 showing long shell units fusing with each other in the lower parts (Scale bar from (B) to (G): 1000 μm). (H) SEM photograph of tangential surface of eggshell from clutch DR8 showing resorption craters (see arrow).

A total of six oospecies were recorded from the study areas (Fig 5). The basic type is spherulitic with discretispherulitic morphotype. The nodes on the external surface are rounded to sub-rounded, separated from each other, and show compactituberculate ornamentation (Fig 5A). The pores on the external surface are sub-circular and pore canals are long and narrow, showing tubocanaliculate pore system.

In *Megaloolithus* oospecies, the growth lines are arched, restricted to shell units, and do not show fusion with growth lines of adjacent shell units. The oospecies *Megaloolithus cylindricus* consists of shell units that are cylindrical, tall, slender, and discrete (Fig 5B). The shell units are short, fan-shaped, broad, and discrete in *M*. *jabalpurensis* (Fig 5C). In *M*. *dhoridungriensis*, the shell units are long, conical, discrete and broader in the upper parts (Fig 5D).

The oospecies belonging to *Fusioolithus* consists of highly arched growth lines that show fusion with adjacent growth lines and shell units are also fused. In the oospecies *F*. *mohabeyi*, the shell units are fan-shaped, large, and fused (Fig 5E), while they are fan-shaped, wide near the base, and fused with a distinct swollen-ended basal cap unit in *F*. *baghensis* (Fig 5F). In *F*. *padiyalensis*, the shell units are slender, irregular, and fused with each other (Fig 5G).

### Petrological studies of host rock

The studied dinosaur clutches occur in two broad areas: Dholiya Raipuriya and Padlya of Dhar District, M.P. In these areas, the titanosaur dinosaur clutch-bearing outcrops occurring in scattered patches are dominantly represented by less than a meter to 4–5 m thick sandy limestone unit that exhibit extensively weathered surface. The freshly exposed surfaces are dominantly yellowish-grey to beige-grey and light-grey coloured. At places, these grey-coloured units laterally pass into red-coloured ferruginous sandstones as in Jamniapura and Padlya areas; elsewhere the latter overlie the former (Fig 4).

**Fig 6:**
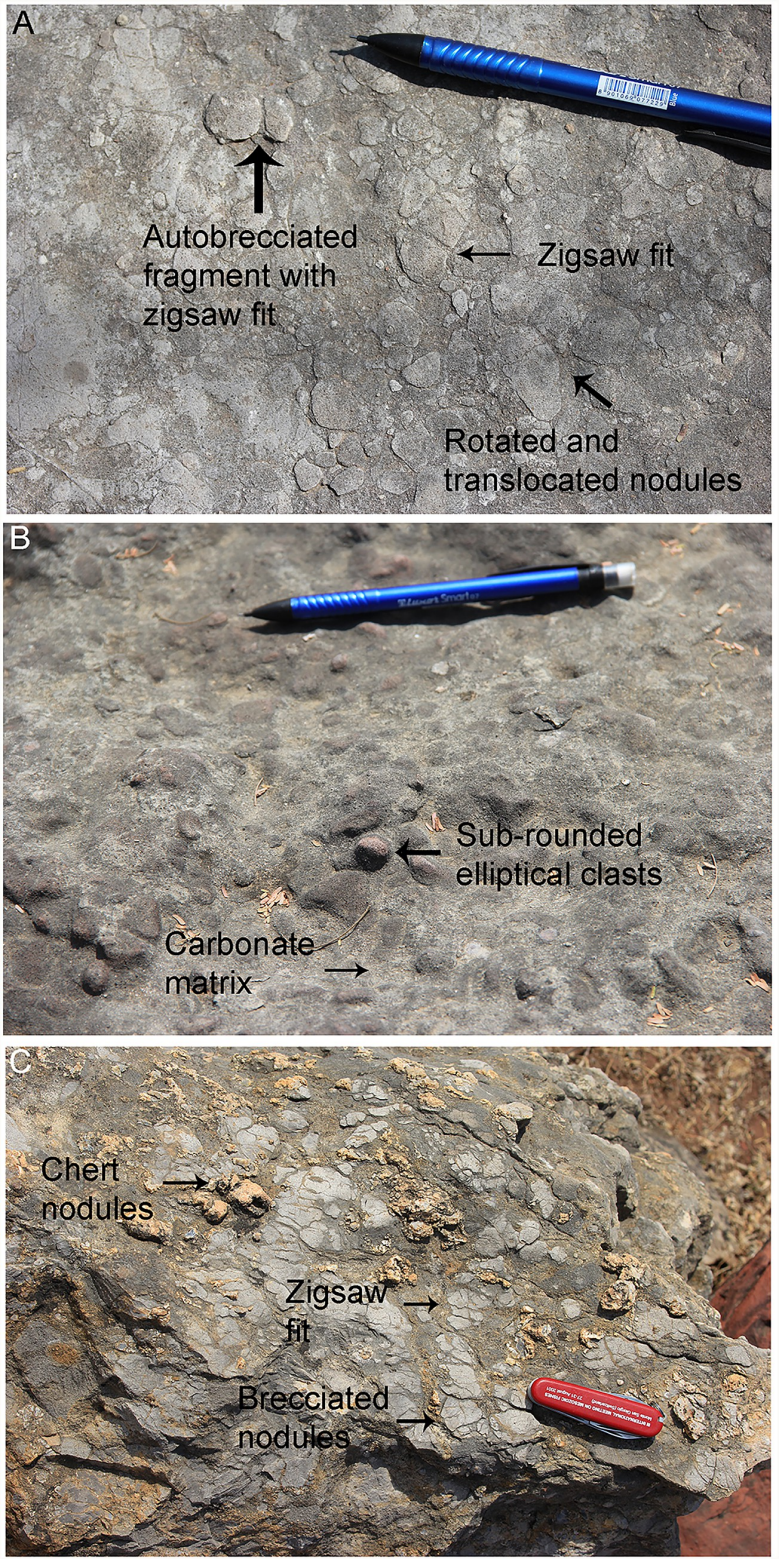
Field photographs showing sedimentary features in the clutch-bearing outcrops. (A) The outcrop from Dholiya Raipuriya shows a characteristic brecciated nodular structure. Some nodules show autobrecciation represented as brecciated fragments with a gap in between them which indicates that the nodules fitted with each other before the disruption. The autobrecciation indicates the non-transported character of the nodules. The areas where zig-saw fit does not exist between nodules indicate their rotation and translocation after shrinkage and collapse (after Dhiman et al. [15]). (B) Intraclast collapse breccias from Dholiya Raipuriya showing variably spaced sub-angular, sub-rounded, and elliptical shaped coarse-grained brecciated clasts in a carbonate matrix. (C) In outcrops at Padlya, the chert exists in association with brecciated nodular limestone where the light grey zones show shrinkage characteristics while the dark grey zones are matrix-rich areas. The brecciated nodules also show zig-saw fit at some places while at other areas the zig-saw fit has collapsed.

The sandy limestone outcrops exhibit shrinkage cracks (Fig 6A and 6C) as is the case of the Lower Limestone unit of the Lameta Formation at Jabalpur [21, 53]. Some outcrops in Dholiya Raipuriya and Padlya also show variably spaced sub-angular, sub-rounded, and elliptical coarse-grained brecciated clasts in a carbonate matrix which were interpreted as intraclast collapse breccia (cf. [21]) (Fig 6B). These outcrops also show chert veins parallel to or cutting across the bedding plane, and chert as nodules or as irregular patches on the surface (Fig 6C).

**Fig 7:**
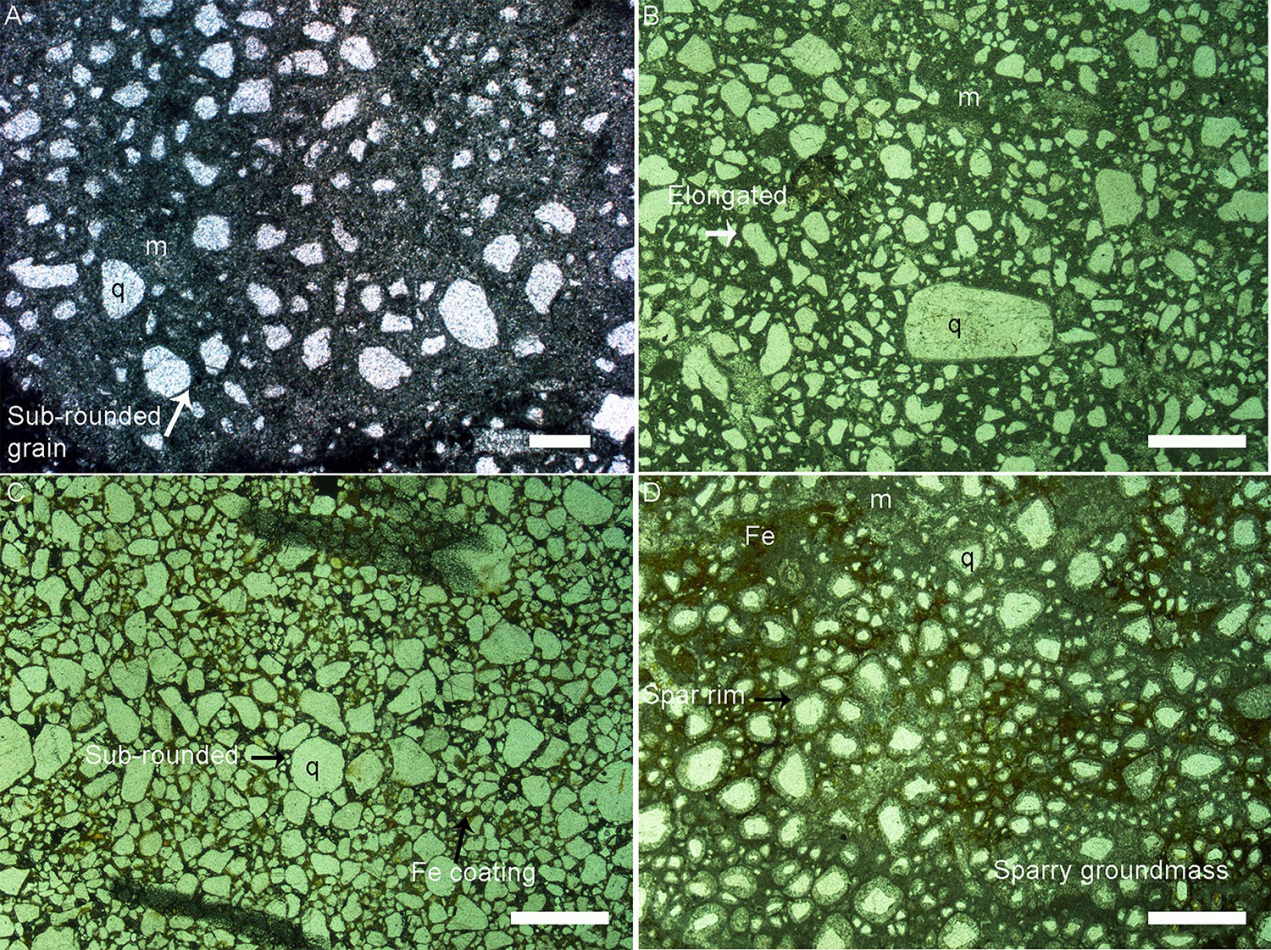
Photomicrographs of thin sections of the dinosaur egg-bearing lithological units. (A) Sandy limestone showing variably sized and shaped quartz grains (q) floating in the calcitic groundmass (m) (Scale bar: 200 μm). (B) Calcareous sandstone with moderate to poorly sorted quartz grains showing angular and sub-rounded grains floating in carbonate matrix; the grains also show elongated shapes. (C) Ferruginous sandstone with a bimodal distribution of quartz grains; elongated grains are present along with round shapes; the iron cement can be seen in the form of reddish to brownish-coloured stains around the quartz grains and pore filling cement. (D) Spar calcite rim and sparry calcite groundmass; the quartz grains are surrounded by corona rims of microspar and in between them dark-coloured, finer grained micrite can be seen (Scale bar in (B) to (D): 1000μm).

The impure carbonates under polarizing light show coarse silt-sized to very coarse sand-sized quartz grains floating in clay-sized carbonate matrix (micrite). The rocks consist of mixed carbonate-siliciclastic lithofacies with varying proportions of siliciclastic material as compared to carbonate sediment. They are classified as sandy limestones (with higher micrite content) and calcareous sandstones (with higher clastic material than carbonate groundmass) depending on the relative percentage of carbonate and siliciclastic constituents (Fig 7). The red-coloured rocks such as in Padlya show red staining due to iron oxide cement and consist of quartz grains, absence of carbonates, and are therefore classified as ferruginous sandstones (Fig 7).

They dominantly exhibit moderate sorting which may grade into patches with poor sorting in the same thin section. The grains are randomly oriented with bimodal to polymodal size distributions. The mineral grains consist of quartz, very few mica grains, and lithic fragments that consist of chert, along with non-clastic material in the form of chemically precipitated carbonates. Detrital, subangular to subrounded, medium to fine-grained, moderately sorted quartz grains showing undulose extinction are present. The grains are floating in a fine grained micritic carbonate groundmass. Carbonate cement occurs in the form of coarse crystalline spar enveloping around quartz grains and as finer micrite to microspar mass in the pore spaces (Fig 7D). Spar also exists in the cracks and veins. Veins of microcrystalline quartz can be observed.

## Discussion

### Titanosaur affinities

On the basis of microstructural studies of the collected eggshells, shape of eggs, clutch patterns, and physical features observed from the clutches, we identify the studied clutches with those of titanosaurid sauropods that have previously been reported from a number of Lameta outcrops in central and western India [3, 4, 6, 7, 9–13,15]. The thickness of the studied eggshells varies from 0.4 mm to 3.2 mm which conforms fairly well to that of Indian sauropod oospecies (0.9–4.8 mm, respectively) [14]. Within the parataxonomic framework, the studied eggshells conform to microstructural features reported from the eggshells of the Upper Cretaceous deposits of Argentina, France, and Morocco [10, 54]. The Argentinian sites (Auca Mahuevo) show fossils of embryos preserved within the eggs which belong to the titanosaurids [55].

The egg clutches of present study show a number of structural features, such as almost intact circular egg outlines, fragmented/morphed egg outlines, eggshell fragments as random deposits and/or present inside and/or outside the egg outline, bottom surfaces of eggs, hatching windows, shell fragment pile/collapsed egg/double bottom, pathological eggs, compressed eggs, isolated-unhatched eggs, remnants of egg material, bowl-shaped eggs, unusually shaped eggs, linearly spread eggshells, and different clutch patterns, such as circular type, combination type, and linear type. These clutches are randomly spaced with respect to each other. Such observations of the investigated sites compare significantly well with other Cretaceous nesting sites, such as the *Megaloolithus* clutches of the Upper Cretaceous Tremp Formation in Coll de Nargó, Spain where the clutches consist of irregularly spaced hatched and unhatched eggs with spacing in between them, intact eggs with broken outlines, shell fragment pile and double bottoms, a few cases of linearly arranged eggshells, besides showing effects of diagenetic alteration [23, 24].

Resorption craters are found in some of the eggshells (Fig 5H), and are very less in number as compared to the clutches showing evidences of hatching. The lack of such resorption craters may indicate either infertile eggs or death of embryo prior to ossification either because of environmental or biological reasons [56, 57]. Additionally, high levels of diagenetic alteration in the form of silicification and recrystallization observed in the extracted eggshells may have obliterated resorption craters. In most of the cases, the extracted eggshells were thoroughly embedded in the rock matrix which may have resulted in the removal of these craters upon diagenesis. However, in clutches of Padlya, where many clutches show intact circular egg outlines and spherical complete eggs, the absence of resorption craters may indicate that these eggs were either biologically infertile, buried too deep causing embryo asphyxiation or affected by environmental events such as flash floods that could have suffocated the embryo much before ossification [6].

The pore canals generally show angusticanaliculate and/or tubocanaliculate pore systems (Fig 5B and 5G). As non-destructive methods are not available, it is difficult to visualize a picture of entire network of pore canal system, and thin section photographs reveal only very few areas where pore canals can be seen in a cross-section. Apart from this, complete eggs are not documented from every clutch which puts constraints on determining clutch type on the basis of eggshell porosity and egg mass [58]. Previous studies have determined covered nests for sauropods on the basis of high eggshell porosity and water vapor conductance studies [55, 59, 60], an indication that megaloolithids usually had maintained a high moisture exchange rate [58].

### Indian oospecies diversity

The dinosaur eggs from the present study belong to two oofamilies Megaloolithidae and Fusioolithidae [11, 61] and six oospecies: *M*. *cylindricus*, *M*. *jabalpurensis*, *M*. *dhoridungriensis*, *F*. *baghensis*, *F*. *mohabeyi*, and *F*. *padiyalensis*. These oofamilies have been considered to have affinities with sauropod dinosaurs [62, 63]. So far three taxa of titanosaurs *Jainosaurus* cf. *septentrionalis*, *Isisaurus colberti*, and Titanosauriformes indet. have been recorded from the Lameta Formation (see [17]). Therefore, the high oospecies diversity does not correspond to the low diversity deduced from skeletal material. However, such a large diversity of oospecies from the present study areas indicates that the diversity did exist in the sauropod taxa of the Lameta Formation [10]. So far, it has not been comprehended as to why megaloolithid eggshell shows so many differences in the radial texture as evident in different shapes of shell units and orientation of growth lines. Such patterns of radial texture could be an artefact of taphonomy where pressure due to sediment pile changes the egg shape and size and hence affect the micro-structure of the eggshell. However, the alignment of growth lines with arching of shell units, placement of pore canals and pore spaces, and bumping nodes on the outer surface points more to the biological nature of the varied morphology of the eggshells. If such differences are biological in nature, then it should have some relation to the activities of mother titanosaur prior to egg laying or more possibly to species diversity.

### Clutch types

**Fig 8:**
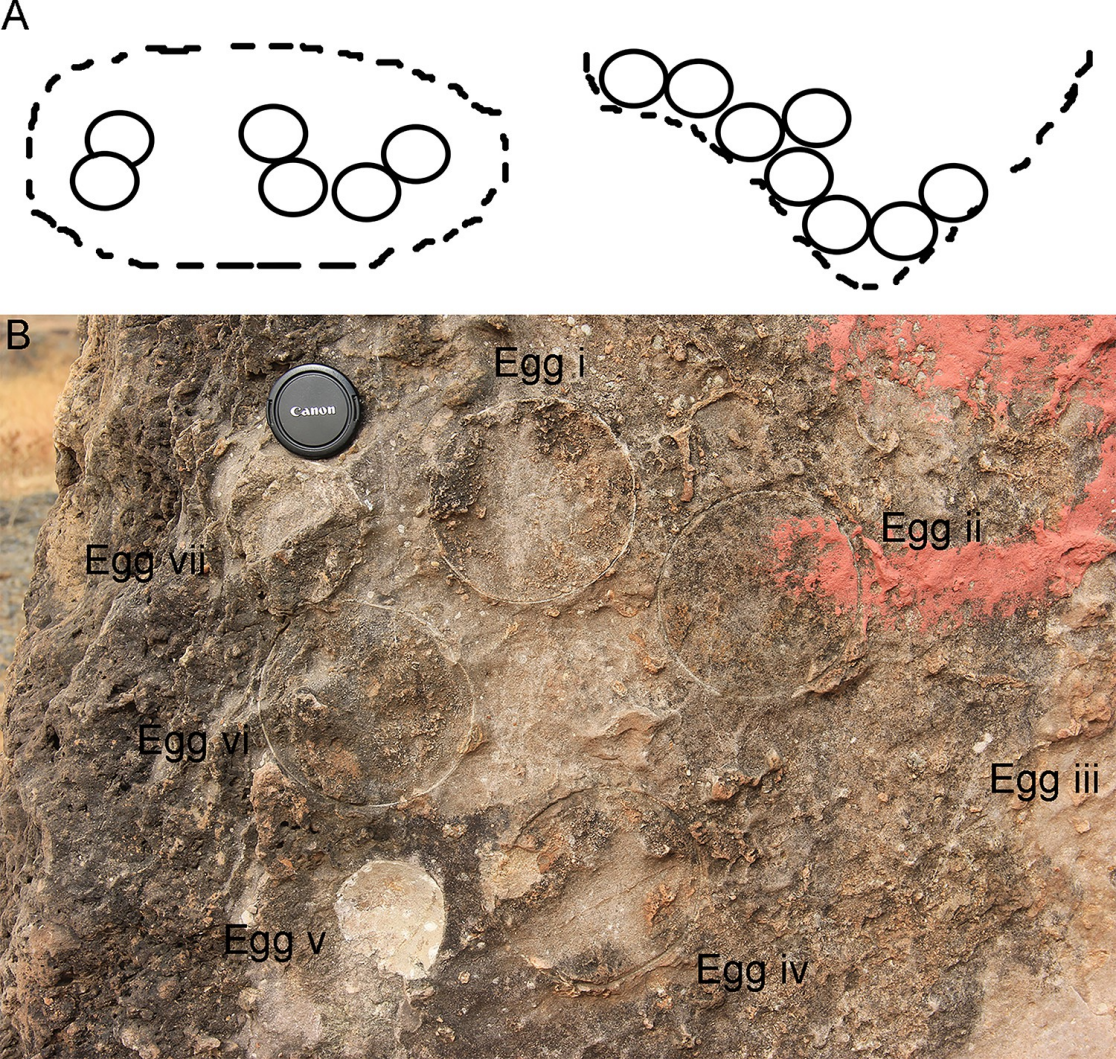
Circular clutch type observed in the investigated areas. (A) Sketch of the circular clutch type (modified after Moratalla et al. [64]). (B) Field photograph of circular type clutch showing eggs with sediment gaps from clutch P35 from Padlya, M.P.

Three types of clutches have been documented from the study areas. The first and the most dominant clutch type is designated as a circular type, in which eggs are randomly distributed in a pit and hence the shape appears conical in cross-section with eggs showing different diameters [64] (Fig 8). This clutch type has been previously reported from the titanosaur nests of Spain, Argentina, and France [23]. Such clutches show around four or more than four eggs closely spaced with respect to each other, with variable diameters, in a plan view. This indicates that these eggs were laid in a pit-like structure with sediment material in between them [64].

**Fig 9:**
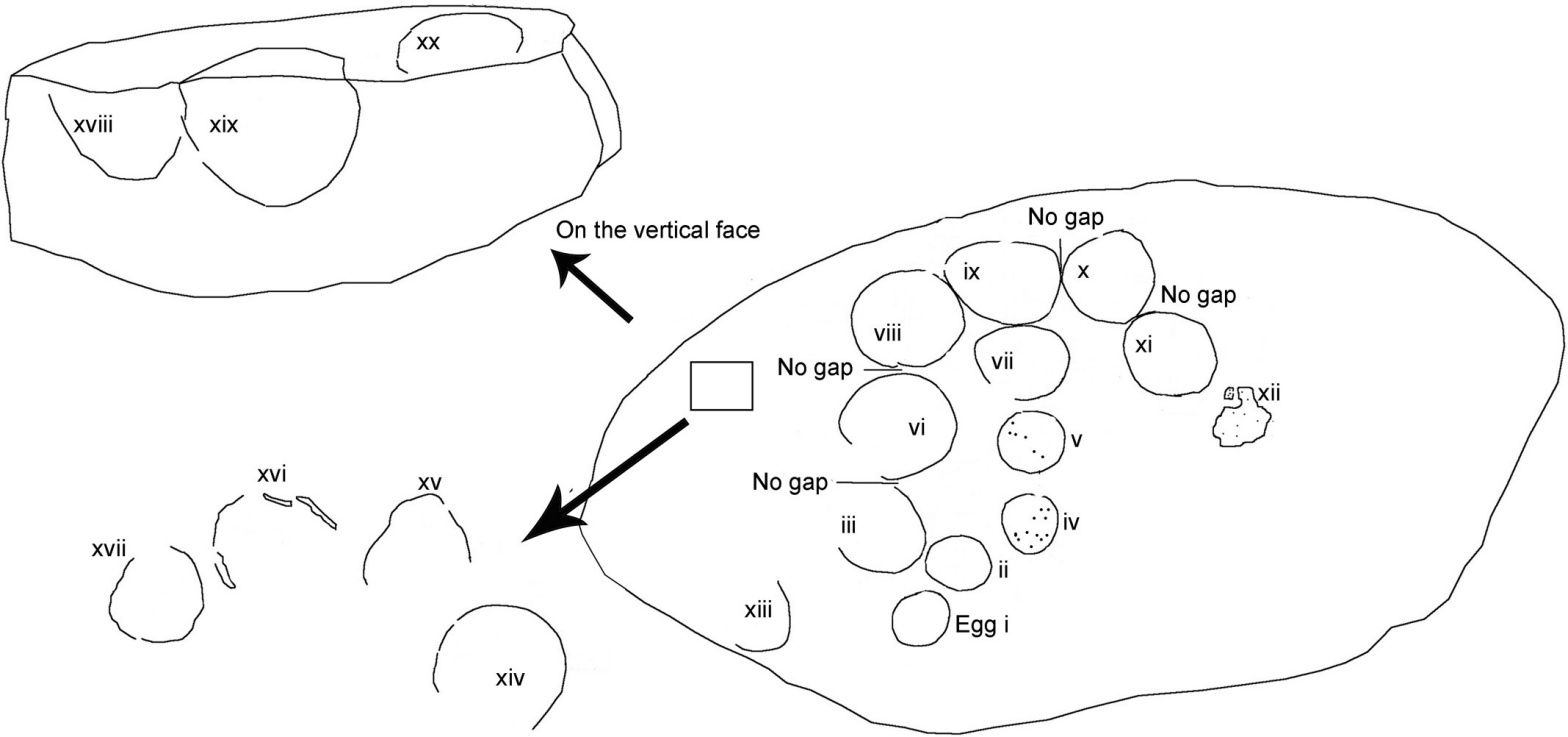
Schematic diagram of clutch P1 from Padlya, M.P. showing evidence for 20 eggs. The eggs i to xii show closely grouped eggs while other eggs in the clutch (xviii to xx) are spaced at a distance from these grouped eggs.

**Fig 10:**
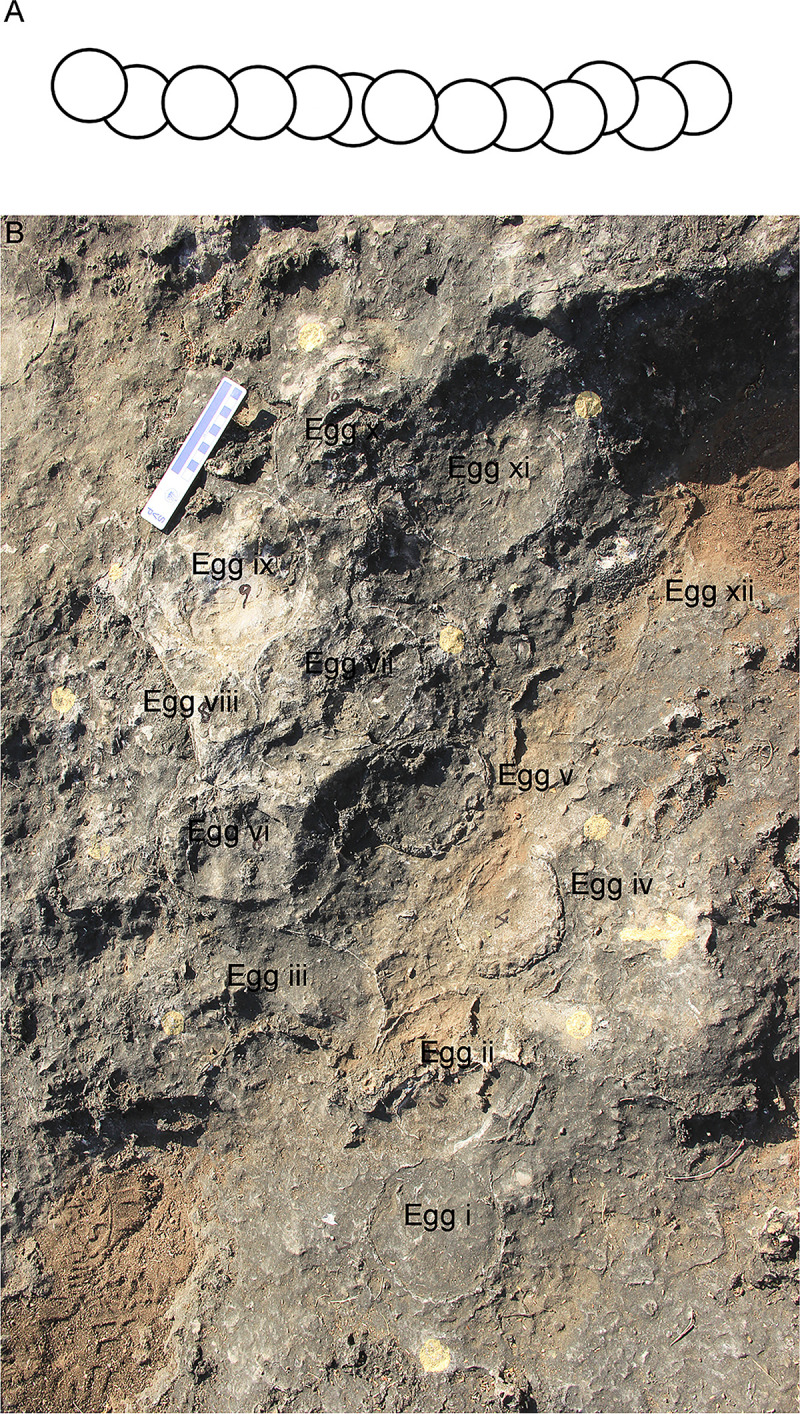
Combination type clutch documented from the investigated area. (A) Sketch of tightly grouped eggs (modified after Vila et al. [24]). (B) Field photograph of clutch P1 showing tightly grouped eggs i-xii in plan view with very little to no spacing between them.

The second type of clutch documented is called as a combination type in which tightly grouped eggs occur along with sediment spacing with respect to other eggs [24] (Figs 9 and 10). The tightly grouped eggs show almost similar diameters indicating that they were possibly buried together in a shallow pit such that their surfaces closely touched each other.

**Fig 11:**
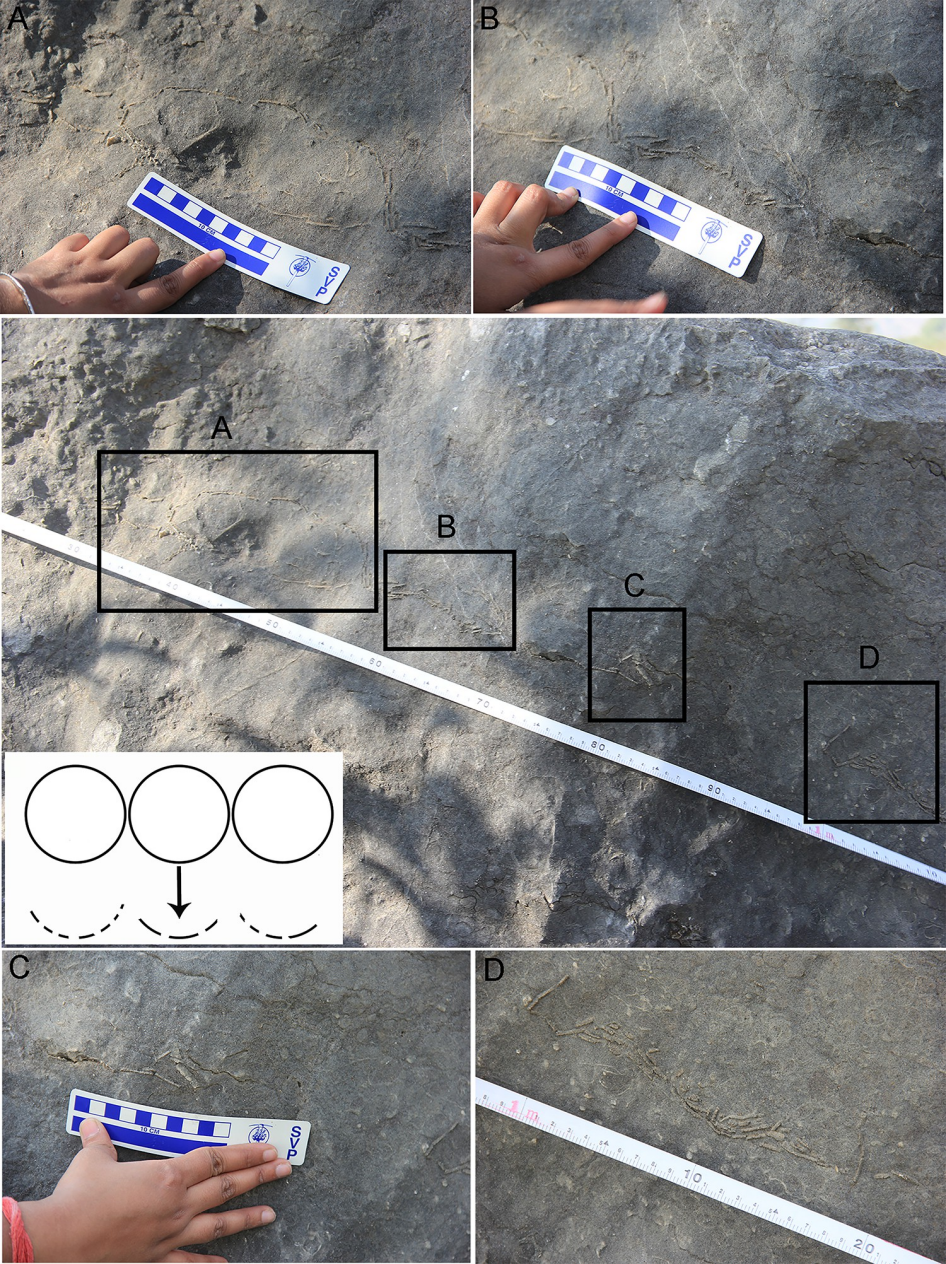
Field photographs of linearly arranged eggs in clutch DR8 from Dholiya Raipuriya. The sketch of such an arrangement is shown in the inset. The eggshell spread starts with a compressed unhatched egg (Box A) which is followed on the right side by concentrically arranged eggshells (Box B) with more eggshells after a gap of around 5 cm to the right (Box C). After another gap of 20 cm, more concentrically arranged eggshells are found (Box D). It appears that around four eggs were laid side by side with respect to each other. The eggshell deposits are concentrically arranged while no such concentric eggshells could be seen in the compressed egg. This indicates that except for this unhatched compressed egg (Box A) other eggs may have hatched, because, if post-burial compression was the reason behind the breaking of eggs, it would have affected all the eggs. The sediment gaps indicate that the eggs were buried in a pit with sediment gaps in between them.

The third type of clutch is known as a linear type in which eggs are distributed in a linear manner such that they lie adjacent to each other in a line [64] (Fig 11). Linear nest type, more common in theropod nests [64], has also been widely reported from sauropod clutches [4, 6, 23, 24, 56, 65–68]. This pattern is documented from Dholiya Raipuriya and the eggs were most possibly arranged linearly and after breakage would have appeared concentric and arc-shaped in cross-section. We assume that because of burial and compressive forces, the eggs would have stretched and taken up linear orientation.

### Egg shape and size

The megaloolithid eggs belonging to titanosaur sauropods have been found to be of spherical and sub-spherical shapes with maximum diameter ranging between 12 and 15 cm [57, 63, 64, 69]. Because of sub-spherical shape, the diameter opposite to the largest diameter may be 10% to 20% shorter [69]. Some titanosaur eggs have been reported to have a diameter as much as 28 cm and elliptical shapes which could be a result of sedimentary compaction, fracturing, and expansion/shrinkage of calcitic sedimentary matrix in which the eggs are hosted [6, 69]. The consequent outline of the spherical eggs seen in a cross-section is circular to sub-circular in the studied clutches.

The diameter of the undeformed and unhatched spherical eggs from the study area falls within the range of 15 to 17 cm. There also exist some undeformed egg outlines which show diameter within the range of 10.5 to 19 cm. Values up to 18 cm have been reported by Mohabey [9] for megaloolithid eggs documented from the Lameta Formation of Balasinor, Gujarat and Mueller-Töwe et al [70] for French and Spanish *Megaloolithus* eggs. The maximum diameter recorded is close to 20 cm which is known in partially elliptical-shaped eggs which have been compressed by post-burial processes. The small values of less than 10 cm observed in the egg outlines (7–3 cm) may actually represent bottom surfaces of eggs preserved in the clutch, opposite diametrical value of the compressed egg, or eggs buried at different depths.

### Number of eggs

**Fig 12:**
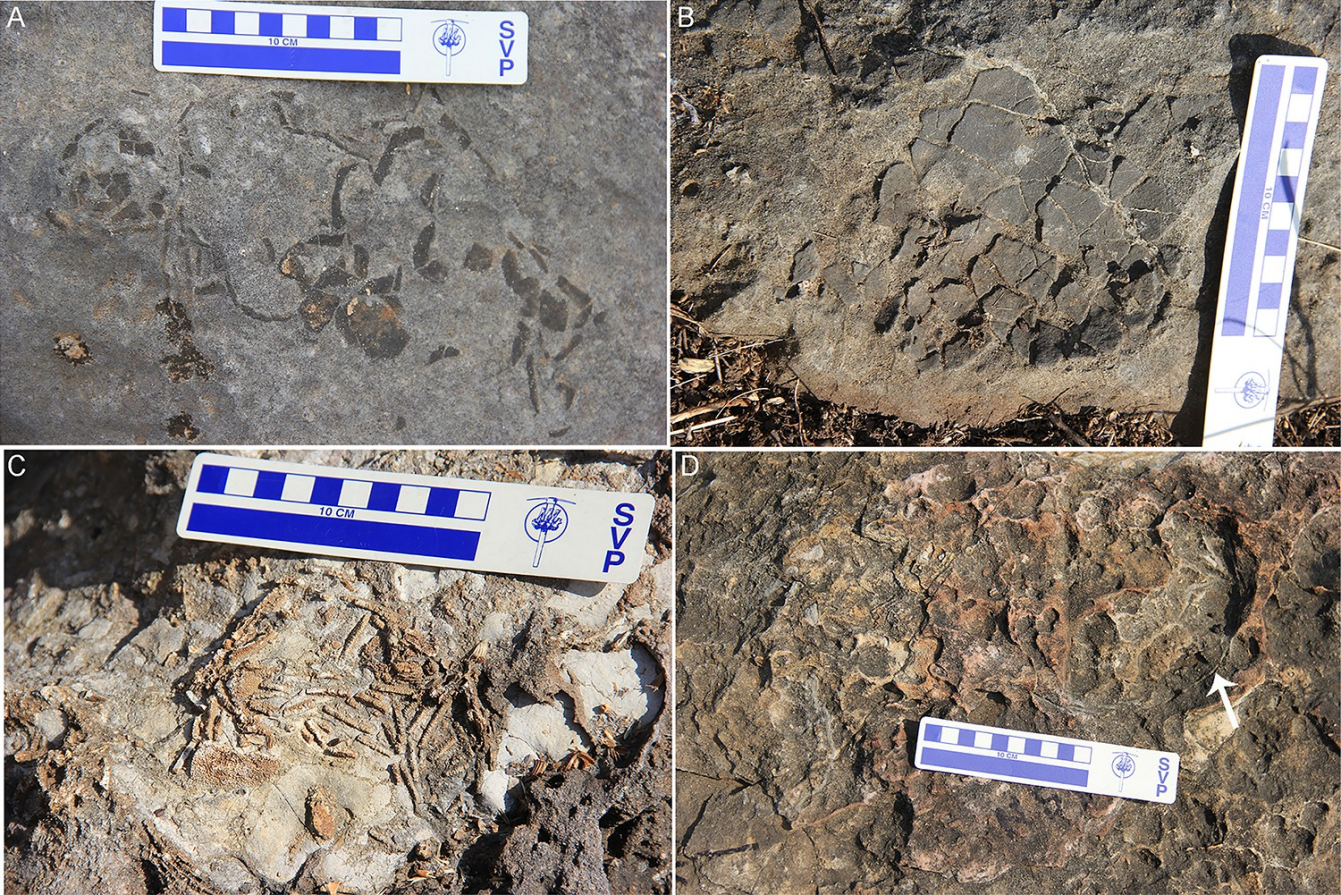
Field photographs showing egg material variably preserved. (A) Fragmented and morphed egg outlines of the eggs from clutch DR9. (B) Egg bottom surface preserved as partially crushed egg bottom in clutch DR4. (C) Eggshells associated closely with each other in the clutch P20. (D) Weathered surface obliterating the eggshell material (see arrow).

The total number of eggs recorded from the nests is 256 and they have been recognized in the form of intact spherical eggs and egg outlines (Figs 8B and 10B), fragmented eggs (Fig 12A), egg bottom surfaces (Fig 12B), and an approximate value has been calculated from eggshells (Fig 12C). The number of eggs documented from the egg-bearing rocks from the study areas range from one to as many as 20 eggs.

Sander et al. [23] suggested that the nests with a low density of eggs are the result of high rate of weathering which eroded other eggs or due to incomplete exposures. The clutches of the Lameta Formation show prominent weathering effects in the field (Fig 12D) and thin section observation of the eggshells also point to this (Fig 5B–5G). Thin sections of eggshells retrieved from the clutches show replacement by silica and calcite recrystallization.

Nests with high density of eggs ranging in number from 20–40 have been identified from the Cretaceous sauropod nesting sites of Río Negro, northern Patagonia, Auca Mahuevo, and Catalonia [24, 57, 63, 71, 72]. According to these studies, 25 eggs represent a typical megaloolithid nest size and nests less than this may represent eroded remnants of much larger nests. Hence, for example, with clutch P1 showing highest number of eggs (20 eggs), we suggest that most of the other clutches must have undergone weathering as evident from replacement by chert leaving just some remnants of the egg material on the surface.

### Hatching window

**Fig 13:**
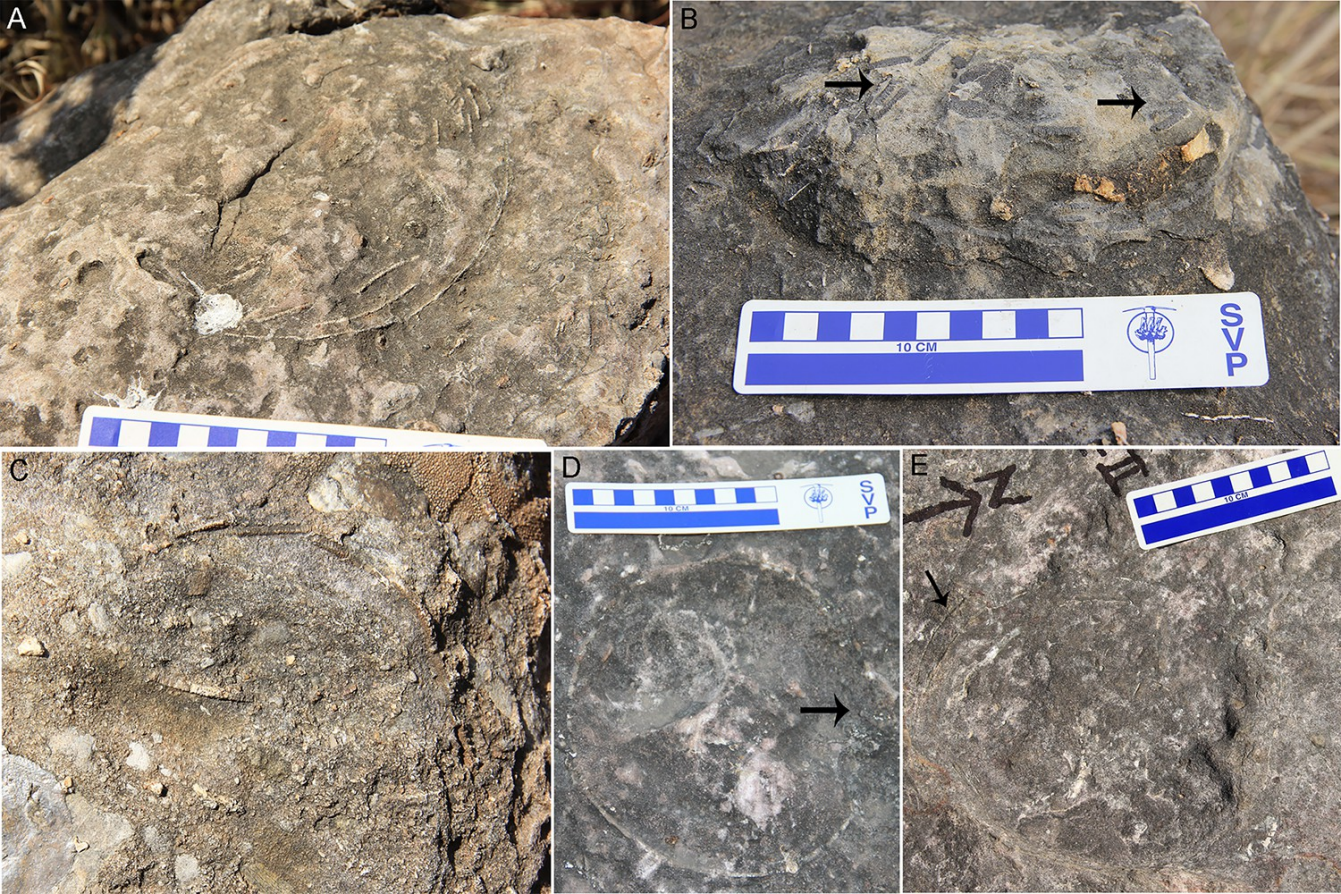
Field photographs showing hatching window and shell fragment pile. (A) Hatching window from clutch P14 present as the topmost eroded portion of the egg and egg fragment piles occurring inside the egg. (B) Egg outline in clutch DR2 with fallen eggshells inside (see arrows). (C) Curved eggshell layer from clutch P20 which is either a remnant of hatching window or due to erosion. (D) Half-preserved egg outline from clutch P5 with little to no eggshells around, indicating it to be either hatching window or erosional remnant. The arrow shows the gap. (E) Egg from clutch P25 with double bottom (see arrow).

Some eggs show a missing upper surface from where the hatchling might have emerged out of the egg (Fig 13A). This has been designated as hatching window [70, 73]. They have been reported previously from many localities [24, 70, 74, 75].

Cousin et al. [73] found eggshell fragments lying with their concave side up at the bottom of the egg and suggested that the eggshells slid inside rather than falling. Such eggshells inside the egg with a hatching window could also be a result of post-burial compression which might have pushed the upper surface of the eggs inside [76]. These openings may not be observed when they are present on a section parallel to the bedding plane [24]. However, if an egg is seen rounded in cross-section but has several eggshells present inside the egg outline, it may indicate that the hatching window is present behind the planar section [73] (Fig 13B).

Expansion of gases accumulated within the eggs produced by the decay of organic matter may also create such openings [77]. Infertile eggs of birds and crocodiles are known to contain such cavities upon burial [78]. Existence of an egg tooth, a hard structure on the beak of embryo bird and jaw of embryo reptile to perforate the egg, has also been suggested as the possible cause for the presence of hatching window inside the egg [79]. The hatchling could have used the egg tooth to perforate the egg and while leaving the egg would have pushed this cap towards the egg bottom [70].

**Fig 14:**
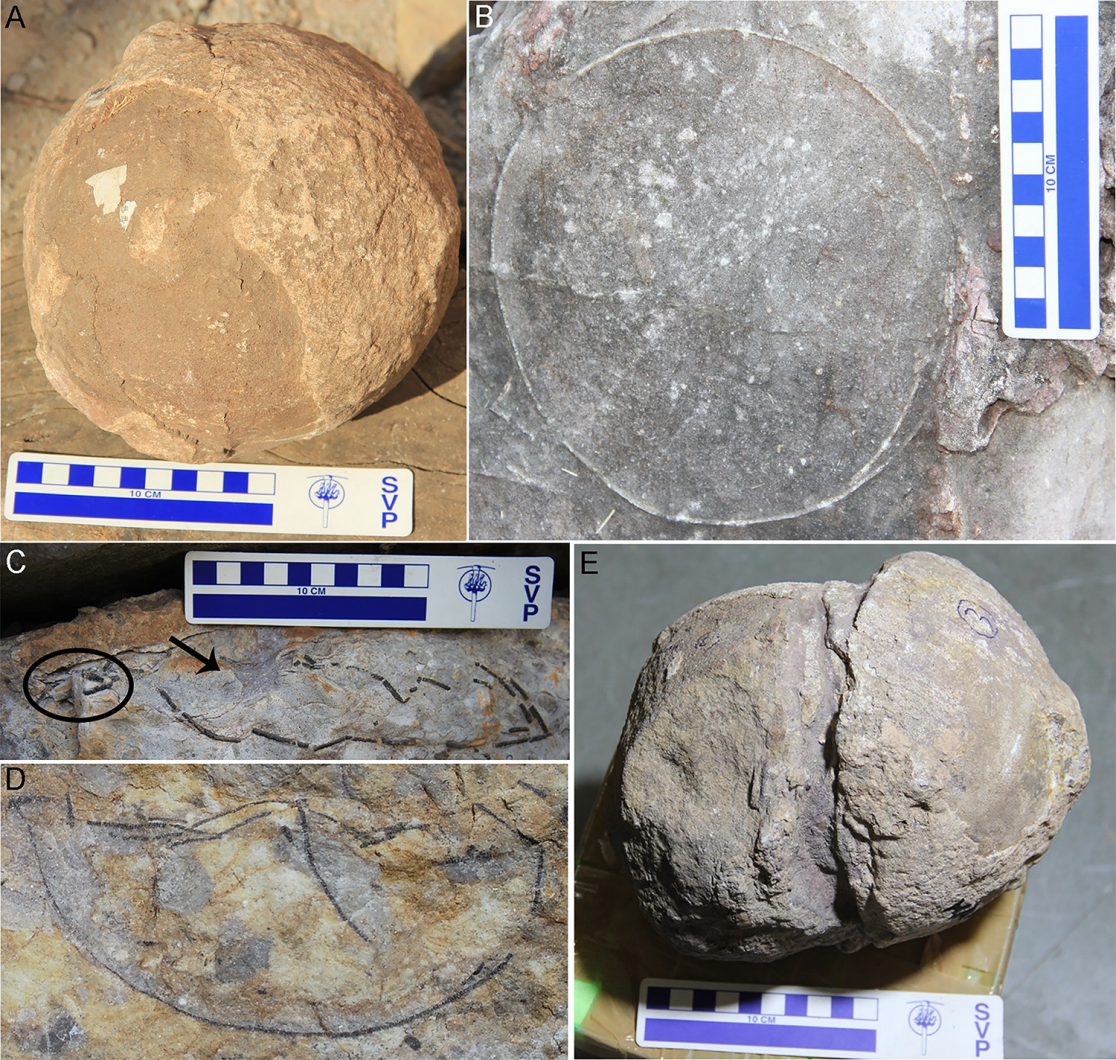
Field photographs of eggs and egg outlines showing various features. (A) Completely unhatched egg from the clutch P43. (B) Almost fully intact circular outline of egg possibly indicating it to be unhatched and no loose eggshells are found in the clutch P6. (C) Compressed egg from clutch DR10 showing hatching window (arrow showing gap) and few eggshells collected just around the hatching window (circled) which possibly represent the remnants of hatching window. (D) Egg from clutch P26 showing curved outline. (E) Deformed egg from clutch P30 showing egg surfaces slipping past each other.

The evidence of the removed eggshell layer of this hatching window was not found during the current study (possibly removed by taphonomic processes or unexposed and is shown as a gap). However, eggshell fragments have been found occurring along the periphery of the egg in the form of broken eggshells (Fig 14C) or accumulated inside the egg in the form of randomly oriented eggshells (Fig 13A and 13B). In some cases, remnants of egg material are present in the form of curved eggshell layer, either intact or broken, in the vicinity of the egg (Fig 13C). Such remnants could represent the removed layer of hatching window. It should be kept in mind that some observations could be misleading because of preservation bias that shows the gaps as hatching window, which may represent an eroded portion (Fig 13D). These windows are known to exist on the upper side of the eggs [70, 73]. The clutches are found from scattered blocks and not from vertical stratigraphic section due to which it is difficult to map out the lower or upper stratigraphic surfaces of the block. Moreover, computed tomography could not be conducted since these clutches are embedded in big blocks of rocks preventing their observation in three dimensions. Hence the possibility of these hatching windows to be remnants of erosional activities cannot be discounted.

### Eggshell fragment pile

Shell fragments at the bottom of the egg have been reported from many localities [70] and also from the studied sites of the present work. The accumulated shell fragments at the bottom of the egg have been named as collapse structure [6], double bottom [73], and shell fragment pile [70]. This kind of distribution may have resulted from hatching, predation, or collapse due to sedimentary overburden. Many clutches show collapse structure/shell fragment pile (Fig 13A and 13B) and double bottom structure (Fig 13E).

Sahni and Khosla [80] described collapse structures resulting when eggshells fall in the egg bottom upon burial under sediment load producing a concentric arrangement of the eggshells. For hatching to be the reason, the eggs need to be already covered with sediments [23]. Cousin et al. [73] suggested that the infant pushed the window outside which subsequently moved along with the sediment matrix into the egg through movements of infant’s limbs. There are also examples where egg is half preserved in outline but there are no or very few eggshells at the bottom of the egg, except in some of these cases very few eggshell fragments are present around the periphery suggesting that the cap was pushed away from the egg and remained outside the egg (Fig 14C). Due to lack of hatchling bones, it is difficult to say whether the concentric arrangement of eggshells is because of slipping inside of hatching window or a consequence of overburden pressure.

### Buried clutches

Sauropod nests were inferred to have been laid either in vegetation mounds [68, 81, 82] or buried in a pit [23, 24, 58, 69, 72–74, 76, 82–85]. Burial behaviour has also been supported by water vapor conductance studies [57, 59, 83, 84, 86], except for Patagonia, Argentina nests that show evidences of an open nest [63]. Tanaka et al. [75] suggested that the mound nests constructed from organic rich sediments used microbial respiration to incubate the eggs and preferred fine-grained pedogenic sediments, whereas the in-filled nests were laid in non-pedogenic, coarse-grained sediments and used solar radiation/geothermal heat for incubation. The latter observation fits well with the studied sites, where clutches have been documented from non-pedogenic, coarse grained sandy limestone to calcareous sandstone lithologies.

Hechenleitner et al. [87] pointed out that in the presence of little vegetation cover and light colour of the sandy sediment, the solar radiation would be rendered ineffective by limiting its invasion to greater depths. This observation fits especially well with the clutches of Padlya where lithology dominantly contains higher content of sandy material (quartz) and preserves greater number of unhatched eggs as compared to other clutches. Similar matrix content inside all of the eggs may indicate that they were laid together as a clutch [75] (Figs 8B and 10B). Additionally, some eggshells within an egg are oriented in such a way that they show gap in between themselves and also between eggshells and egg bottom which indicates that eggshells fell inside the egg along with sediment material (Fig 13B). This also attests to the fact that the eggs were buried [23, 24, 67, 76, 88].

Another helpful interpretation by Mueller-Töwe et al. [70] is that the eggshells occurring within the egg bottom and not around the eggs are strongly supportive of underground incubation and hatching. Many clutches show gaps in between the eggs of variable diameter (Fig 8B). Sander et al. [23] suggested that a loose packing of eggs is possible because after laying a few eggs they were covered with soil/plant materials followed by deposition of more eggs; had they been put in a pit without any material in between them, they would have touched each other and the eggs at the centre would have been at the lowest level making the eggs show slightly variable diameters. Since we have not found many evidences of plant remains we assume nesting in a shallow pit as a more suitable nesting habitat of titanosaurs of the studied areas. Higher number of documented clutches may also support that they were laid as subsurface clutches as it increases the preservation potential [64]. The clutch P1, showing many eggs touching each other, must have been buried together as a group in a shallow pit (Figs 9 and 10).

### Unhatched eggs

Isolated unhatched eggs have been excavated from Jhaba and Padlya that give a clear indication of their being either biologically infertile or death of embryo before hatching because of environmental factors (Fig 14A and 14E). These eggs may also have been buried quite deep that would have led to suffocation of the embryo. Pathological eggs are also biologically incapable of hatching. Additionally, there are many egg outlines that are circular to sub-circular in shape and lack any eggshell fragments either around or inside the egg (Fig 14B). Such eggs have been assumed to be unhatched.

Akhada and Dholiya Raipuriya sites show more evidences of broken eggs than Padlya. In Akhada no complete egg has been found, while in Dholiya Raipuriya there has been only one case of unhatched egg, in the form of a compressed egg (Fig 11A). In Jhaba and Padlya, four and twenty clutches, respectively, provide evidence for unhatching in the form of pathologic eggs, unhatched eggs, and partially intact outlines with no eggshells in the vicinity. In Padlya, the number of hatched and/or damaged eggs is more than unhatched eggs. Apart from those eggs that show clear evidences of pathology and hence remained unhatched, some environmental factor may have been on work that rendered the non-pathologic eggs to remain unhatched. These eggs must also have been laid close to lake/pond margins and occasionally got submerged.

### Compressed eggs

Eggs from the studied sites show compression which has resulted in a sub-circular to elliptical shape of these eggs (Fig 14C). Such compression is caused by lithologic compaction as a consequence of which the eggs may get diametrically altered and also suffer fracture of the egg surface [23, 69]. In few cases, the overburden pressure has resulted in two halves of the eggs slipping past each other (Fig 14E). This could have resulted because of egg fracture and further displacement most possibly after the egg was filled with sediment which prevented crushing of the egg. Had it been fractured before being turned into a hard sediment in-filled egg it would have a pile of shell fragments around it. The egg must have remained stable and undamaged after burial for a long period of time.

An unusual shaped egg has been documented with an inwardly curved eggshell layer that slumped in the middle (Fig 14D). Similar kinds of eggs have been described by Mueller-Töwe et al. [70]. As eggshell behaves as a brittle material, Mueller-Töwe et al. [70] suggested that the egg achieved flexibility because of decay and leaching processes. These authors have also associated eggshell softening with decalcification which is related to embryo ossification. We also suggest that after internal soft material of the egg was decayed, the topmost layer started to settle down, however for this to have happened the egg should not have been buried at deeper levels and not had much sediment infilling. Additionally, this egg material observed from a plan view can be a taphonomic artefact where arrangement of the eggshells shows a curve like feature. It is important to note that a complete curved line shown by eggshells inside a complete egg as reported by Mueller-Töwe et al. [70] is not observed during the present study.

### Pathological eggs

**Fig 15:**
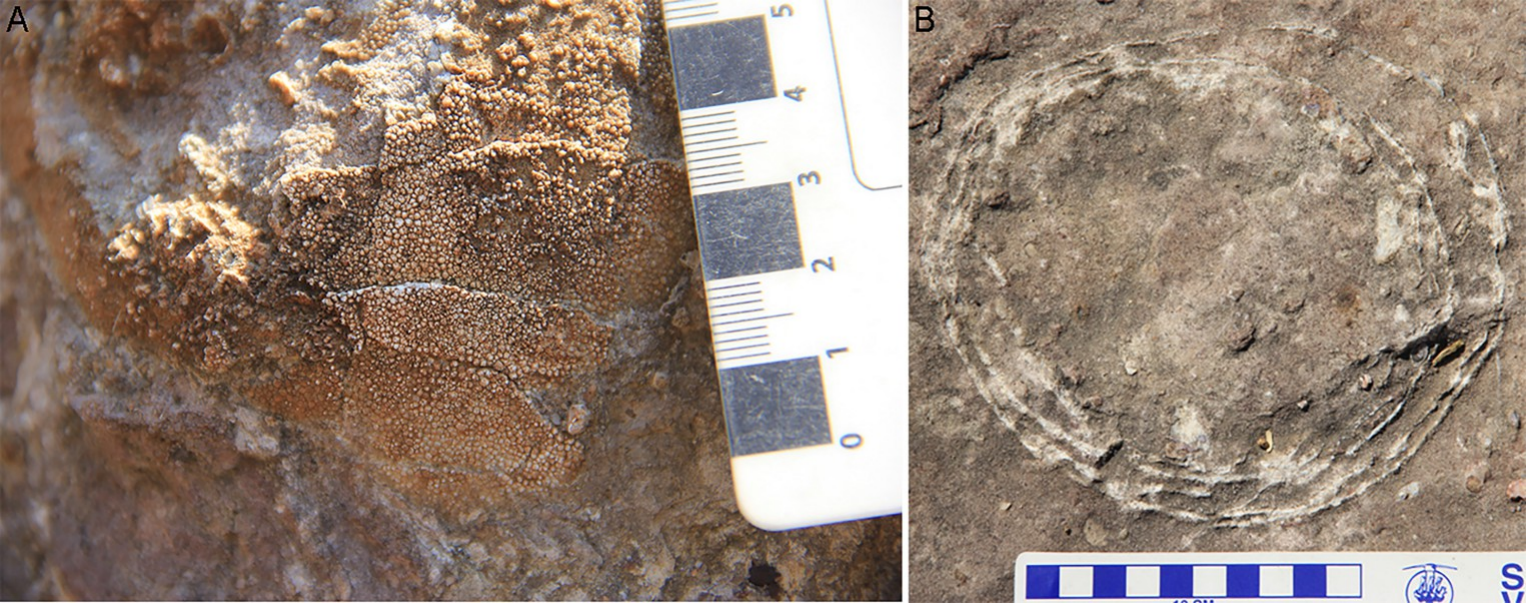
Pathological eggs documented from the Lameta Formation. (A) Multi-shelled egg pathology as shown by double eggshell layers from the clutch P20. (B) Ovum-in-ovo pathology as revealed by gap in between two complete eggshell layers from the clutch P7 (after Dhiman et al. [51]).

Pathological eggs have been documented from clutches of Padlya. Clutch P20 upon a closer inspection show 2–3 superimposed eggshell layers (Fig 15A). Clutch P7 shows an unusually-shaped egg consisting of a gap between two completely round eggshell layers with another broken layer in between (Fig 15B). Cousin et al. [73] opined that the enclosed layer could be a result of fallen hatching window inside making it look like the double bottom structure. However, good preservation of an egg-like shape in P7 implies the inability of the egg to hatch because of infertility caused by pathology. The first type of pathology of P20 is interpreted as a multi-shelled egg, while the second type of P7 is identified as an ovum-in-ovo condition (Dhiman et al. [51]).

An ovum-in-ovo pathologic egg forms when a completely formed egg is pushed back up the oviduct or towards magnum through muscle contractions where it comes across an unshelled egg which is devoid of albumen. Later both these eggs move to the shell producing region where they get shelled [89, 90] or the egg which has moved back up the oviduct undergoes deposition of yolk, albumen, and shell membrane if the next ovulation has occurred [91], or the completely shelled egg with its yolk stays back till the unshelled egg comes and then both get shelled [89]. A multi-shelled pathologic egg forms when a completely formed egg remains in the shell producing region and as a consequence it gets shelled again [90]. A reptile while retaining the entire clutch in the oviduct may push the last egg back up the oviduct through muscle contractions causing it to get shelled again [57, 90]. Ewert et al. [92] suggested that the multi-shelling happens by oviductal retention of eggs of one clutch that are re-shelled when the next clutch is prepared to get shelled. So, the multi-shelled egg is considered to have been laid at the bottom of a nest as it would be the first one to be released [92]. In both pathologic cases, the egg stays infertile and fails to hatch as the blocked pore canals do not allow passage of gases thus suffocating the embryo [90]. Such abnormal eggs result when the parent is facing physiological and/or environmental stresses, such as lack of nesting sites, floods or droughts, high population, sickness, diet, etc. [57, 91, 93, 94].

### Reproductive biology

The finding of ovum-in-ovo pathologic egg from titanosaur opens up possibility of sequential egg laying pattern in these titanosaurs [95]. The aves possess a specialized uterus and release their eggs one by one while amniotes consist of a generalized uterus and the eggs are laid together as a clutch [90]. Although the crocodiles and alligators have a specialized segmented uterus with separate regions for shell membrane and calcitic shell deposition similar to birds, their pattern of egg laying is reptilian [90, 96]. With the documentation of ovum-in-ovo egg from titanosaur, it becomes probable that the oviductal functional morphology of this group of dinosaurs was similar to birds making them capable of sequentially laying their eggs [51]. However it should be kept in mind that the clutch pattern of titanosaurs which shows eggs randomly spaced with similar matrix content inside and outside the eggs, indicates their nesting pattern to be more similar to crocodiles. These observations indicate that the reproductive biology of sauropod dinosaur is more similar to that of archosaurs (crocodiles, birds) than to non-archosaurian reptiles [95].

### Parental care

It is considered that if hatchlings were altricial they would have remained in the nest for a long period of time, pointing to parental care and would have caused more breaking of the eggs. Such instances have been observed in *Maiasaura* nests [97]. Breakage of a nest as one of the factors to vouchsafe for parental care may lead to wrong interpretation in our case as parental care has been considered to be non-existent in sauropods because of the size differences between juveniles and adults and closely spaced clutches [64, 98]. Moratalla et al. [64] suggest that unhatched eggs and lack of records of juvenile bones point towards a precocial behaviour and a lack of parental care. Absence of hatchlings may indicate the precocial behaviour of the hatchlings implying that they left nests quite soon after hatching [80]. Sander et al. [25] suggested that since the clutches of Auca Mahuevo were laid quite close to each other, trampling of hatchlings would have been possible. Dholiya Raipuriya clutches are more closely spaced than Jhaba and Padlya clutches and the former shows more evidences of damage to eggs. Alternatively, they could have been simply hatched eggs as they show good evidences of hatching and were not affected by any pathology. Some eggs have only bottom surfaces preserved which show partial crushing (Fig 12B). Either they are the result of erosion, or sedimentary compaction, or the result of pressure exerted by juvenile once it hatched and moved out of the egg [99].

### Colonial behaviour and site fidelity

Unlike in other Cretaceous deposits, where dinosaur clutches have been documented in a vertical stratigraphic section [21, 73], the studied clutches of Bagh-Kukshi areas are found in scattered and detached outcrops of the Lameta Formation. In these areas, outcrops showing a stratigraphic section showing the stratigraphic position of the Lameta Formation with respect to pre-Lameta deposits and overlying Deccan Basalts is not preserved, such as the sections seen in Jabalpur, Kheda District, Nagpur, Chandrapur District, and Salbardi [4, 21, 49] except for a site close to clutch P7 inside DFNP. In the latter case, the Nodular Limestone of the Bagh Group underlies the Lameta Formation (Fig 3). In the absence of a well-developed vertical section, it is difficult to assess the number of nesting cycles, site fidelity, and colonial nesting behaviour [23]. It is also difficult to say if all the eggs in a clutch were laid at the same time or not.

Colonial nesting behaviour has been previously reported for sauropods [23, 63, 88, 98, 100]. It is a preferred choice in ecological communities as it provides safety from predators [75, 101, 102]. Colonial nesting is indicated by extensive clutches and morphologically similar eggs [6]. The presence of different oospecies in the studied clutches may indicate that same nesting area was shared by different sauropod taxa [72, 102, 103]. Modern turtles and birds which are known for colonial nesting behaviour show close spacing between the nests [23]. Few of the closely spaced Jhaba and Padlya clutches may indicate adoption of a colonial nesting behaviour. Akhada and Dholiya Raipuriya are quite localized areas with 5 and16 clutches, respectively, closely spaced with respect to each other. They may indicate a colonial nesting behaviour as all of the clutches show some evidences of either hatching or damage to eggs. However, such closely spaced clutches must have prevented manoeuvring and parental care. It can also be interpreted that perhaps these closely spaced clutches were laid at separate time intervals, but if the sites had been revisited by sauropods, they must have recorded trampling of clutches by sauropod feet considering their huge size. Either the site was reused or surface topography with slightly different elevations existed on which clutches were laid [6]. However, it is difficult to know for how long site reuse might have lasted. Additionally, it may very well be considered that many of the clutches are unexposed while others may have suffered removal because of erosion. Considering the good preservation and large number of clutches, it can be suggested that the studied areas were quite favourable sites for laying of eggs.

Nesting sites have been documented from calcareous sandstone to sandy limestone lithology which is similar for most of the Indian dinosaur clutches reported from the Lameta Formation [6, 21]. It can be assumed that this specific lithology was the preferred one for laying of eggs. Modern crocodiles tend to prefer nesting habitats closer to water sources [104] and titanosaurs nesting behaviour seemingly similar to that of crocodiles led us assume that sauropod dinosaurs may have preferred similar habitats. The soft sediments closer to water sources such as rivers or lakes must have been used for partial burial of the eggs [6]. In the case of Indian nesting sites, these soft sediments were covered by Deccan lava flows leading to their extensive preservation [6].

### Other fossil remains

The nesting sites are unusually lacking in the body fossils of adults, juveniles, and embryos of sauropods. There are many sedimentary deposits from around the world where both osteological and oological remains are found in the vicinity of each other [55, 105, 106], including Indian sites such as Bara Simla Hill of Jabalpur and Balasinor of Gujarat [6]. Lack of adult sauropod bones from the area where oological remains are found could be either due to dominance of erosional processes or selection of the locality for the purpose of laying of clutches and not for habitation [106]. It could also be possible that the bones are unexposed and remain to be documented.

Sahni et al. [6] suggested early and deep burial of eggs, water loss because of high porosity in a dry environment, and pedogenic modifications to be the reasons for lack of embryos and hatchlings. The absence of fossils of juveniles may also point that they left the nest soon after hatching [6, 70] indicating a precocial juvenile.

### Depositional environment

The medium to coarse grain size and subangular and subrounded shape of quartz grains along with moderate sorting observed in thin sections of egg and eggshell-bearing rocks indicate a short distance of transport (Fig 7A and 7B). The groundmass dominantly consists of micrite with few occurrences of spar cement (Fig 7D). The precipitation of calcium carbonate can take place in situ either by precipitation aided by organisms or evaporation of surficial or groundwater [107]. In the absence of fossils, we deduce dominantly chemical precipitation to be the reason behind carbonate formation. In the case of palustrine carbonates (explained below), this microcrystalline calcite precipitates in a lacustrine body which then emerges sub-aerially to form palustrine carbonate [108]. Many broken grains show matching edges with spaces filled by calcite indicating primary origin of calcite under the influence of shrinkage and accompanying autobrecciation (cf. [21, 53]). Chemically precipitated silica occurring as replacement of calcite is evidenced on the basis of chert-filled veins, random spots of silicification, and veins of microcrystalline quartz.

**Fig 16:**
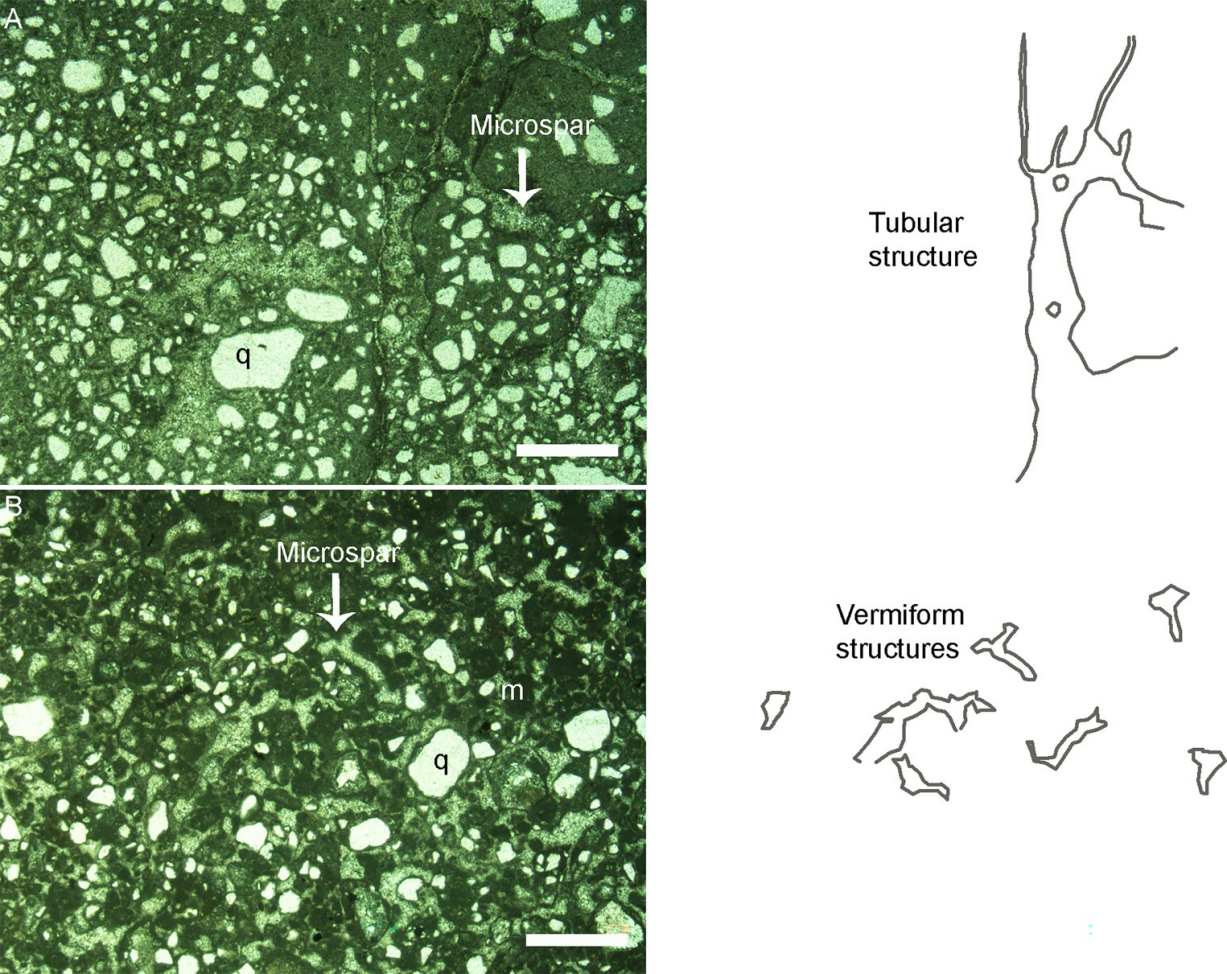
Photomicrographs of sandy limestone showing alveolar-septal fabric (accompanied by sketches). (A) The alveolar fabric from Jamniapura, Dhar District consists of a tubular structure filled with microspar and the lower part shows quartz-rich zones, the in-between dark zones inside the tubular structure could be residual micrite. (B) The alveolar fabric from Dholiya Raipuriya, Dhar District shows wispy and vermiform structures consisting of micro-spar replaced micrite channels and networks irregularly spaced, with micrite groundmass as dark patches (Scale bar: 1000 μm).

Alveolar septal fabric has also been documented from few thin sections (Fig 16). It is a network of small, adjacent voids and microchannels made of micrite walls and pores consisting of sparry calcite and quartz grains and is characteristically associated with the presence of root traces [109]. These are common in palustrine deposits [109].

Barely any rock unit was documented that consisted of only micrite and laminations, hence there are no evidences for the development of profound lake deposits as was previously observed in the Lower Limestone of the Lameta Formation of the Jabalpur area [53]. Palustrine environment occurs close to those lakes which have a very gentle slope such that the environment of the wet alluvial plain merges with that of lake margin producing a marshy environment where fauna and flora flourish in a wet soil environment [110]. These have been defined as shallow and freshwater deposits that show evidence of both subaqueous deposition and subaerial exposure [111]. The shallow gradients extensively expose the marginal areas when lake levels fall [112]. They form in low gradient and low energy marginal lacustrine areas, temporary ponds isolated between siliciclastic sediments, and peritidal settings [108, 113, 114].

In palustrine deposits, the events of desiccation and water-logging alternate because of periodic fluctuations in water level which causes reddish-coloured mottling in the zones closer to lake shores. Areas at higher levels show more red horizons because of greater exposure [111]. Several sandy limestones in Jamniapura and Padlya region exhibit such reddish colours and consist of dinosaur eggshells. Additionally there are many exposures of other red-coloured rocks in Padlya and Jamniapura which are in fact ferruginous sandstones with iron oxide as cement. In Padlya, these exposures interfinger with the grey coloured units (Fig 4). This can be considered as a transition from palustrine to alluvial setting. Those palustrine limestones that show reddish hue may have undergone high levels of oxidation upon exposure. In places where ferruginous sandstone and sandy limestone outcrops occur in isolation but in close association (Fig 4C), they represent palustrine carbonate deposits along with alluvial/floodplain deposits [108]. Thin sections of these rocks show moderate sorting similar to those of the river sediments [110]. However, in the absence of sedimentary structures pointing to the presence of channels, it is difficult to validate this observation.

It has been found that the presence of plants at the lake margins act as clastic filters hence trapping of terrigenous sediments takes place [115]. We have not found any direct evidence of plant material in the studied sites (though fossil wood logs are known from other sites of the Lameta Formation, such as Bagh-Zeerabad area) which can be considered as one of the reasons behind the presence of high amount of clastics in some of the deposits. Apart from this, a sediment-laden sheet flow can deposit clastics in lakes [110]. This sediment-laden flow upon entering the lake experiences a sudden fall in velocity, deposits coarse sediments at the river mouth, and forms a delta [110]. The clastics were mainly deposited by smaller channels and distributaries into the wetlands and ponds [107]. Such quartz rich deposits have also been considered to occur in regions having vegetation and affected by low relief, and hot and humid climate [116].

In the absence of any evidence for marine conditions, it is considered that chertification happened as a post-depositional process through siliceous solution [117]. The silica might also have been derived from the overlying Deccan Traps [118]. Silica rich waters are capable of replacing calcium carbonate with diagenetic chert [110]. Secondary chert forms due to diagenetic silicification and generally occurs as nodules which coalesce to form layers [110]. The clutch-bearing units must have served as an aquifer where groundwater collected which would have been enriched in calcium and bicarbonate with silica, the latter being possibly derived from the coeval as well as the overlying Deccan Traps.

The palustrine deposits are characterized by features such as mottling, nodules, brecciation, desiccation cracks, reworking of carbonate fragments, and vegetation signatures such as alveolar structures [108, 119, 120]. These features also indicate that the palustrine settings were commonly subaerially exposed because of fluctuations of the shallow lake level [111, 121–123]. Carbonates from the studied areas show the above evidences along with dispersed siliciclastic grains and intraformational clastic grains in micrite-microspar, cracks filled with spar, and microspar crystals around quartz grains similar to the palustrine sequences of the Lower Limestone of Jabalpur [21, 53]. Absence of palaeosol and calcrete indicate short and intermittent intervals of subaerial exposure [109]. A preliminary stable isotope analysis of the host rocks also suggest freshwater palaeoenvironmental conditions and the eggshell geochemistry reveals C3 palaeodiet with rivers and shallow pools as water sources (S1 File).

### Taphonomy of the nesting sites

The large-scale palustrine associations include lacustrine deposits, reworked alkaline flats/pond/lake carbonates, distal alluvial sediments, and evaporites (in saline conditions) [53, 120, 124]. The lack of evaporites from our study area indicates predominantly the presence of freshwater deposits [115] which is also confirmed through preliminary stable isotope studies of the investigated areas (S1 File). Evidence of profundal lacustrine deposits is not observed. The outcrops from the study areas showing compact and buff to yellow-coloured micrite with several evidences of palustrine settings indicate a carbonate flat/pond setting with subaqueous sediment deposition and subaerial desiccation [53]. Sheetwash events can affect these carbonate flats/ponds ([53], see their Fig 11). A large number of scattered outcrops indicate a number of palustrine deposits that were associated with small and shallow lacustrine bodies/alkaline flats/ponds/wetlands. Alluvial deposits, possibly related to a fluvial system existed, especially in Padlya, as evidenced by ferruginous sandstones that resulted in a high amount of siliciclastic material. The presence of intraclastic collapse breccias may indicate the presence of supratidal conditions associated with lake bodies in one particular locality of Dholiya Raipuriya (Fig 6B), however, this observation is limited to a single locality.

The clutches were laid in soft sediments of palustrine areas that got occasionally submerged producing a number of unhatched eggs in Padlya, and these also got subaerially exposed producing desiccation and brecciated surfaces. The subaerial exposure also rapidly cemented these deposits often affected by sheetwash events that transported detritus [21, 80]. This sheetwash flooding also hindered hatching [80]. The floodwaters would deposit a thin layer of sediment which would help in egg preservation along with matrix cementation and may also inhibit hatching [80].

The relatively high water levels caused chemical/biochemical precipitation of carbonates; during subaerial exposure these were affected by brecciation, root action, and desiccation [111]. Those areas that suffered prolonged exposure, such as topographically relatively higher areas, developed palaeosols and calcrete [111]. However, we did not find such deposits in the studied areas. Those areas that were extensively low lying remained submerged for longer periods of time. The alternating events of wetting and drying of carbonate muds caused cracking, dissolution, enlargement of voids, and reprecipitation of carbonates and cements [110]. It is understood that the dinosaurs laid their eggs in soft sandy sediments of palustrine bodies both close and away from the small lacustrine bodies/ponds/wetlands, extensively scattered in central and western India, as previously suggested [6, 21, 53, 125].

**Fig 17:**
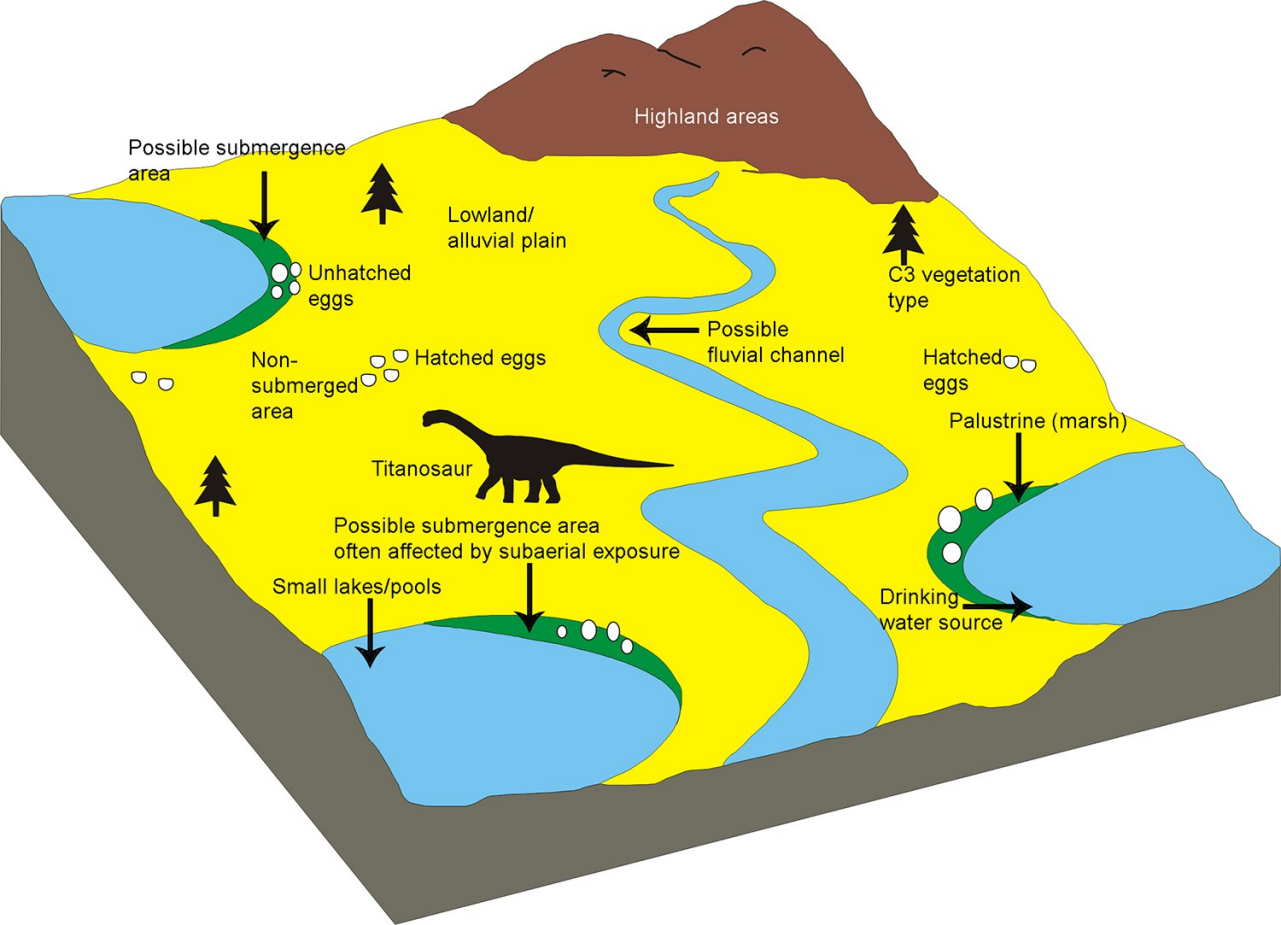
A block diagram showing the interpreted depositional environment of the Lameta Formation in the study areas. It is inferred that some of the clutches were laid close to the banks of the aquatic bodies (lakes/ponds) while others were deposited away from the lakes or ponds. The clutches laid close to the margins were prone to frequent submergence by water and thus got buried under sediment and remained unhatched, while the clutches laid away from the margins could hatch and hence showed more broken eggshells.

The hatching of eggs cannot be completely identified only on the basis of plan and cross-section views of the egg deposits. Those egg outlines that show hatching windows, shell fragment pile, fragmented outlines, and eggshell deposits, are considered hatched, while those egg outlines that show intact outlines are considered unhatched along with the occurrence of isolated-unhatched eggs. Hence the ratio of hatched to unhatched clutches for the sites Dholiya Raipuriya, Jhaba, and Padlya are, 15:1, 14:4, and 32:20, respectively (study sites Akhada and Jamniapura have a small spatial extent and mostly show random eggshell fragments with 1–2 cases of fragmented to partially intact egg outlines). Overall, it appears that the biological reasons that render an egg unhatched were very unlikely as there are only two cases of pathologic eggs. While the hatched clutches of Dholiya Raipuriya must have been laid in palustrine deposits slightly away from the pond margins, the clutches of Jhaba and Padlya that show evidences of unhatched eggs were laid close to pond margins out of which few eggs would have been submerged while others escaped submergence. During times of high magnitude hydrological events, these eggs would have undergone submergence. Frequent submergence would have entombed them in a thick sediment cover. Since the evidences of hatched eggs is higher than the unhatched eggs, very few clutches must have been laid close to the margins, while most of the other clutches would have been laid at conducive higher places for hatching (Fig 17). Out of a total of 92 clutches, only 25 clutches show unhatched eggs which indicate that suitable sites existed in the palustrine flat area for titanosaurs to lay the eggs.

As the ferruginous sandstone do not preserve eggs, it is inferred that the palustrine areas were the most suitable sites for laying of eggs possibly because of softer sediments in marshy zones that would have been easier to excavate by the animal. Study areas of Dholiya Raipuriya and Jhaba were more suitable for nesting as they have evidences of egg hatching, while Padlya had sites that were both suitable and unsuitable for hatching.

In a few cases, the eggs have well-preserved macrostructures in the form of unaltered shape and external surface. The eggshells also show preserved shell units. However, the preservation is poor when it comes to the ultrastructure dealing with growth lines. But the documentation of nitrogen-bearing organic compounds testifies to the fact that macromolecules were preserved [15]. On the basis of the presence of organic compounds, it is concluded that the eggshells were partially unaltered in few cases while most of the other eggshells show diagenetic alteration to a great extent. The stable isotope analysis also helps in supporting pristine conditions of few eggshells (S1 File).

Eggshells are composed of pore canals due to which permineralization effects are rampant in the pore canals which are often filled with silicate minerals. Since the eggshells are originally composed of calcite, in many cases where growth lines have been obliterated, recrystallization of the eggshells can be seen showing different textures. In some cases, the entire original eggshell has dissolved away and silica rich minerals have replaced them.

The calm depositional conditions can be considered as an important factor that enabled preservation of a large number of clutches [126]. Carnivorous predators may have acted as biological agents of destruction thus breaking the eggs prior to hatching or preying on embryos/hatchlings. Even the animals that moved around the clutches would have trampled the eggs. However, the well-preserved shapes of a large number of eggs indicate that the rapid burial because of high magnitude hydro-meteorological events aided in their preservation and protection from biological agents of destruction [126]. Additionally it can be suggested that the nesting area was safe from predators which made it a more suitable choice for both laying of clutches and further fossilization.

## Conclusions

Based on integrated histological, palaeobiological, and taphonomic studies on the titanosaur dinosaur nesting sites of the Lameta Formation of the Bagh District of lower Narmada Valley, we conclude the following:

1. The oological material is found as intact clutches, isolated eggs, and eggshells and have been assigned to titanosaurs on the basis of macro- (circular, linear, and tightly grouped eggs as clutch types, egg diameter within 15 to 17 cm, buried clutches) and micro-structural (compactituberculate surface ornamentation, spherulitic shell units, angusticanaliculate and/or tubocanaliculate pore systems) observations. Six oospecies have been identified: *Megaloolithus cylindricus*, *M*. *jabalpurensis*, *M*. *dhoridungriensis*, *Fusioolithus baghensis*, *F*. *mohabeyi*, and *F*. *padiyalensis* which points to a possible high diversity in Indian sauropod taxa.
2. Hatching windows have been found which indicate spaces from where hatchling emerged out of the egg. In some cases, half-preserved eggs with eggshells inside are present indicating that possibly the hatching window slid inside while the juvenile was moving out. They also indicate that the eggs were partially buried as these eggshells are present with sediment material. This, known as collapsed eggs or shell fragment pile, can also be a taphonomic artefact where sediment burial can push the top surface of egg inside. The hatching window also occurs in the form of closely spaced eggshells just outside the egg outline or concentrically arranged around the egg outline (called as double bottom) when the egg is seen through a plan view.
3. Isolated unhatched eggs indicate infertility, death of embryo prior to hatching, deep burial of eggs, pathology, environmental factors such as floods, or being laid close to lake/pond and/or fluvial channels.
4. Both multi-shelled and ovum-in-ovo pathologies have been documented. The former is common in both reptile and bird eggs, while the latter has so far only been reported from avian eggs. This is the first report of ovum-in-ovo egg in titanosaur eggs further pointing to possibility of sequential egg laying in titanosaurs (Dhiman et al. [51]).
5. On the basis of previous water vapor conductance studies, eggshells inside the egg with spacing, and variably sized eggs with matrix gaps, it is inferred that the clutches of the study areas were partially buried in a shallow pit as is the case with modern crocodilians, and used solar radiation/geothermal heat for incubation.
6. Parental care must have been absent as the size difference between juvenile and parent dinosaur is enormous and clutches are closely spaced. This also supports precocial behavior of juveniles which must have left clutches soon after hatching. On the basis of abundant clutches, closely spaced clutches, similar eggs, and different oospecies, it is concluded that the titanosaurs of the study areas adopted for colonial nesting behavior.
7. Surprisingly, no osteological remains pertaining to embryo, juvenile, and parent dinosaurs have been found. This is perhaps because the dinosaurs did not live where they laid their eggs, or the osteological material is still unexposed or removed by erosion. The eggs are lacking embryos possibly because of their deep burial and modification due to plant root activity. The absence of juvenile skeletons may indicate their precocial behavior.
8. On the basis of the presence of chemically precipitated micrite, floating siliciclastic grains, alveolar-septal fabrics, grainy intraclastic fabric, brecciation, shrinkage cracks, quartz-filled cracks, mottling, and calcitic nodules, a low energy-low gradient freshwater palustrine depositional setting that underwent episodes of subaqueous deposition and subaerial exposure is inferred for the dinosaur clutch-bearing lithounit. This type of palaeoenvironment existed in a fluvial/alluvial setting that received clastics and experienced intermittent oxidation intervals between episodes of floods.
9. The eggs were laid in soft, marshy palustrine sediments associated with small lake/pond bodies. The clutches close to the lake/pond margins would occasionally get submerged thus remaining unhatched. Frequent exposure resulted in desiccation and shrinkage cracks, while during submergence sediments covered the clutches. Since there is a greater number of hatched eggs as compared to unhatched eggs, it appears that few clutches occur close to lake/pond margins (Jhaba and Padlya) while mostly they occur away from the lake/pond margins and hence were hatched (Akhada and Dholiya Raipuriya).

## Supporting information

S1 File S1 Palaeodiet, palaeoenvironment, and palaeoclimate. (DOCX)
