# Supplementary material for: New Late Cretaceous titanosaur sauropod dinosaur egg clutches from lower Narmada valley, India: Palaeobiology and taphonomy
Source: PLoS One. 2023 Jan 18;18(1):e0278242. doi: 10.1371/journal.pone.0278242 (PMC9848018; doi:10.1371/journal.pone.0278242)
Supplement: S1 File — (DOCX) [file pone.0278242.s001.docx]

**S1 Palaeodiet, palaeoenvironment, and palaeoclimate**

Oxygen and carbon isotopes study of pristine eggshells and the host rocks was undertaken to obtain insights on the palaeoclimatic conditions prevailing at the time of egg deposition and preservation, and also to understand the dietary preferences of titanosaur dinosaurs.

From Dhar district, M.P., three eggshells and six micrite samples from host rock have been analysed (Table S1.1). In addition to these, titanosaur eggshells belonging to Kallamedu Formation of Ottakoil, Cauvery Basin have also been analysed for comparative purpose. All the rock specimens, except P16 have isotope values close to each other which indicate that similar kind of material was analysed. Additionally, both eggshells and rock specimens of A3 and P7 show different isotopic values indicating that diagenesis was not significant as that would have caused homogenization of the values (Fig S1.1, Table S1.1). The δ^18^O values of eggshells show more variation as compared to their δ^13^C values (Table S1.1). All of these provide evidence for low diagenetic effects on the selected specimens.

The mean values of δ^13^C and δ^18^O of rock carbonate of Dhar district is –8.5±1.1 ‰ (n = 6) and –8.1±1.0 ‰ (n = 6), respectively (Table S1.1). For eggshells, the mean value of δ^13^C and δ^18^O from Dhar district is –12.3±0.3 ‰ (n = 3) and –4.5±3.3 ‰ (n = 3), respectively (Table S1). The δ^18^O and δ^13^C values of eggshell of Ottakoil from Cauvery Basin are –2.7 ‰ and –11.9 ‰, respectively (Table S1.1).

The δ^18^O and δ^13^C values in this study shows close resemblance to the results reported by Sarkar et al. (1991) where the analysis has been conducted on dinosaur eggshells and host limestones of Lameta Formation from Kheda district of Gujarat (western India), Pisdura and Chandrapur from Maharashtra and Jabalpur from Madhya Pradesh (central India). The authors have reported the δ^18^O values of host limestone to be –8.0 ‰ which is quite similar to the data obtained in present study (–8.1±1.0 ‰) from the micrite drilled from the host sandy limestone of Dhar district (Table S1.1). Upon comparing this value with the previously calculated average Cretaceous freshwater and marine water δ^18^O values (–10.2 ‰ and –4.5 ‰, respectively; Keith & Weber, 1964), it becomes apparent that the δ^18^O values of the host rock falls closer to the δ^18^O values of freshwater than marine waters. On the basis of the δ^18^O values of host rocks in present study area and the results obtained by Keith and Weber (1964) and Sarkar et al. (1991), it can be concluded that the host rock was deposited in a freshwater palaeoenvironment. This is also evident from the depleted ^18^O values which also resemble to freshwater sources (Sarkar et al., 1991). Additionally, the data of nest P16 shows slightly different value of –6.4 ‰ (Table S1.1), which may indicate cementation due to meteoric water (Tandon et al., 1995). The slightly more negative δ^18^O values of –9.1 ‰ of nest DR9 (host rock) may indicate slight thermal alteration effects (Tandon et al., 1995).

In previous study by Sarkar et al. (1991), the δ^13^C values of the host limestone is similar to our observations (–8.5±1.1 ‰; Table S1.1). Upon comparison with the previously calculated average Cretaceous freshwater and marine water δ^13^C values (–4.3 ‰ and +0.25 ‰, respectively; Keith & Weber, 1964), our values are closer to the freshwater carbonate, however it is lower than expected for freshwater isotopic values. It has been suggested that the lower values of δ^13^C of freshwater carbonate is because of the inputs of terrestrial organic matter which upon oxidation produced CO_2_ and the resultant bicarbonate (Sarkar et al., 1991). As per this observation, the δ^13^C values of host rock in the present study area may have had inputs of terrestrial organic matter, which further indicates presence of marshy lacustrine and/or fluvial palaeoenvironmental conditions (Sarkar et al., 1991). Further, the depleted ^13^C values of micrite carbonate of host rocks also suggest that the dominate contribution of organic matter was coming from C3 vegetation (Jha et al., 2020).

The δ^13^C value of the eggshells shows a mean value of –12.3±0.3 ‰. This value falls in the range given by Sarkar et al. (1991) for the dinosaur eggshells of Kheda district and also from Pisdura, Jabalpur, and Asifabad (–10.0 ‰). Additionally, the dinosaur eggshells from Ottakoil also show a similar value to the ones obtained from Dhar district (–11.9 ‰). The similarity in δ^13^C values of eggshells between the Lameta and the Kallamedu formations indicate preference for similar types of diet. Such uniformity in carbon isotopic data of eggshells from both formations indicates similar preference of food for titanosaur dinosaurs, which also strengthens the parataxonomic classification that puts ootaxa from both these formations in *Fusioolithus*. A metabolic enrichment of ~16 ‰ also needs to be taken into account which gives the δ^13^C values of the Lameta Formation and the Kallamedu Formation to be –28.2 ‰ and –27.9 ‰, respectively. The δ^13^C values after correction of metabolic enrichment correspond to δ^13^C values of modern C3 vegetation (Basu et al., 2015). Similar δ^13^C values have also been reported by Erben et al. (1979) for Cretaceous sauropod eggshells of France and Spain. The δ^13^C values of dinosaur eggshells of *Megaloolithus* from the Upper Cretaceous Tremp Formation are –27 ‰ to −29.6 ‰, falling in the range of our data, suggesting dietary preference of C3 vegetation (Riera et al., 2013).

The δ^18^O of the dinosaur eggshells from the Lameta Formation show a range from –8.3 ‰ to –1.9 ‰ and from the Kallamedu Formation is –2.7 ‰. As compared to other isotopic values, it appears that δ^18^O values of dinosaur eggshells shows a higher spread (Table S1.1). However, the spread is not very significant when compared with δ^18^O values of dinosaur eggshells (–8.0 ‰ to +6.9 ‰) measured by Sarkar et al. (1991). Sarkar et al. (1991) have considered the spread in δ^18^O values to be an effect of differences in δ^18^O of source waters from where the dinosaurs consumed water. Moreover, these δ^18^O values may also indicate the δ^18^O value of the leaf water as it has been observed that modern ostriches mostly consume water from plants (Montanari et al., 2013). The low δ^18^O values may indicate a fluvial source originating from relatively higher elevations which usually has depleted ^18^O precipitation, while high δ^18^O may indicate lacustrine source with small and confined pools of water which suffered evaporation due to semi-arid conditions (Sarkar et al., 1991). However, since the variation in our data is not significant, the source may have been accumulations of water pools in floodplains impacted by sheetflows/floods frequently. As we do not see any positive δ^18^O values, the water bodies in the vicinity of our studied areas were not significantly evaporated (Tandon et al., 1995; Tandon & Andrews, 2001).

More recently, Kumari et al. (2021) have suggested humid palaeoclimatic conditions for the Lameta Formation of Jabalpur on the basis of morphological, micromorphological, and geochemical analysis of the palaeosol facies of the Mottled Nodular Bed. On the other hand, semi-arid palaeoclimatic conditions have been previously deduced for the Lameta Formation of Jabalpur on the basis of stable isotope studies, pedogenic structural features, and palaeolatitude position (Brookfield & Sahni, 1987; Tandon et al., 1995). In the Lameta Formation of Balasinor area, similar climatic conditions prevailed and have been suggested through stable isotope studies of dinosaur eggshells (Sarkar et al., 1991). Stable isotope geochemical analysis performed in the present work also points to semi-arid palaeoclimatic conditions. Moreover, in semi-arid palustrine sequences, extensive vegetation is lacking because of periodic high salinity or alkalinity along with evidences for extensive rooting and pseudomicrokarsts (Platt & Wright, 1992). However, brecciation related to desiccation polygon formation and related desiccation features are present along with evaporites (Platt & Wright, 1992). In humid palustrine sequences, evaporites are absent and desiccation features are very less but root structures and microkarst are in plenty (Platt & Wright, 1992). On the basis of the lack of extensive vegetation with only scarce preservation of alveolar-septal fabrics, lack of evaporites, and abundance of shrinkage features, the Lameta outcrops of the present study area are interpreted to have experienced semi-arid to sub-humid climatic conditions and no humid conditions can be inferred as suggested by Kumari et al. (2021) from the Jabalpur region. Moreover, Dzombak et al. (2020) have suggested that stable palaeoclimatic and palaeoenvironmental conditions existed before and after the Cretaceous-Palaeogene (K-Pg) extinction on the basis of palaeoclimate proxies, Floral Humidity Province proxy, macrofloral records, and sedimentology. The authors suggest that the climate remained arid only for some time and was not semi-arid in a climatological sense. The previous works have also suggested rise in humidity levels during the initiation of K-Pg followed by drier conditions (Dzombak et al., 2020).


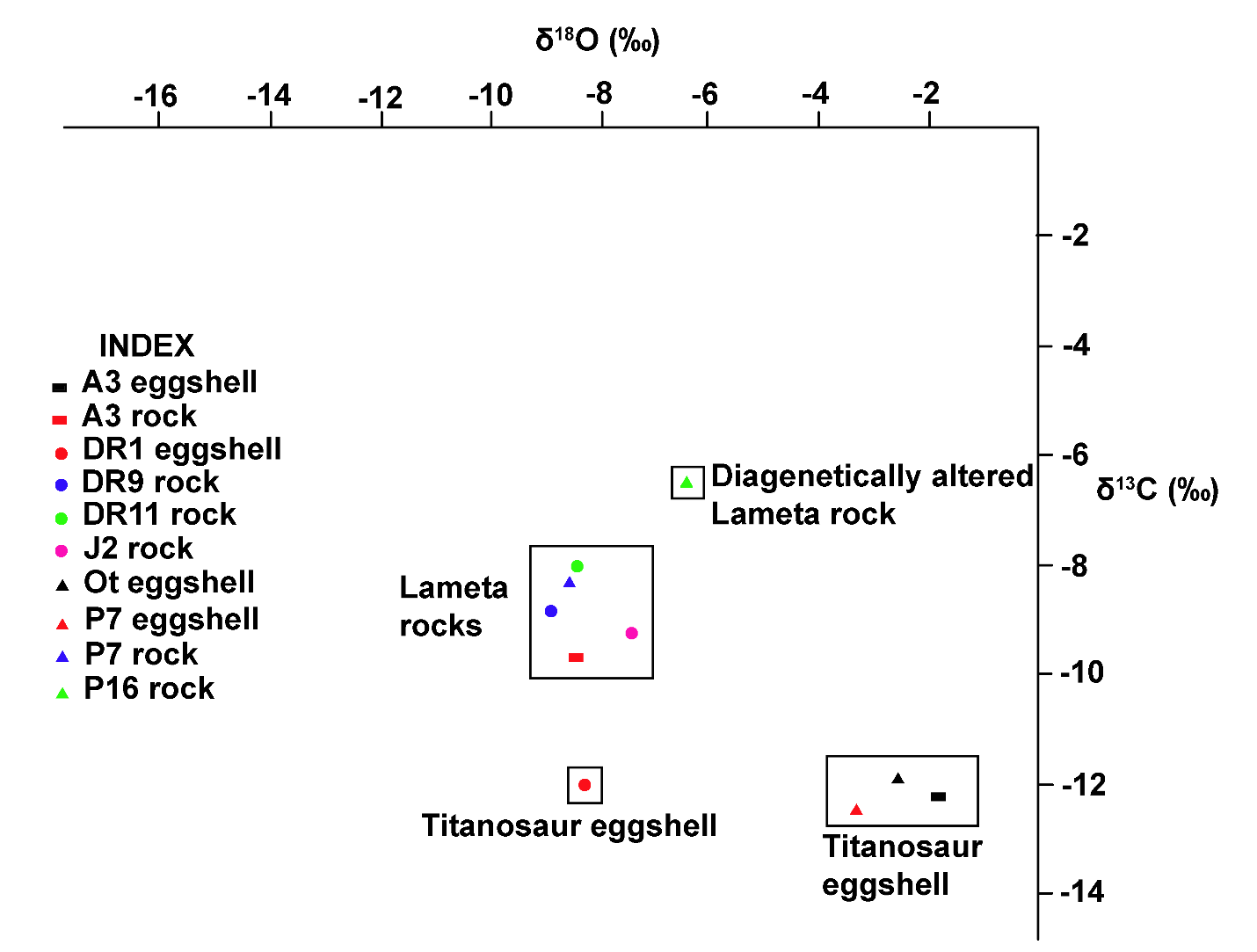


Fig S1.1 Stable isotope data of δ^13^C and δ^18^O of dinosaur eggshells and host micrite from the Lameta Formation of Dhar district and dinosaur eggshells from the Kallamedu Formation (A-Akhada, DR-Dholiya Raipuriya, J-Jhaba, O-Ottakoil, P-Padlya)

Table S1.1 Stable isotope data of δ^13^C and δ^18^O of dinosaur eggshells and host micrite from the Lameta Formation of Dhar district and dinosaur eggshells from the Kallamedu Formation (Rep stands for Repeat analysis; A-Akhada, DR-Dholiya Raipuriya, J-Jhaba, O-Ottakoil, P-Padlya).

| **Locality** | **Specimen name** | **Specimen type** | **δ^18^O (‰)** | **δ^13^C (‰)** |
| --- | --- | --- | --- | --- |
| Dhar District, Narmada Valley, Madhya Pradesh, India | | | | |
| Akhada | A3 | Eggshell | - 1.9 | – 12.3 |
|  |  | Sandy Limestone | – 8.4 | – 9.8 |
| Dholiya Raipuriya | DR1 | Eggshell | – 8.3 | – 12.0 |
|  | DR9 | Sandy Limestone | – 9.1 | – 8.7 |
|  |  | Sandy Limestone_Rep | – 9.0 | – 8.9 |
|  | DR11 | Sandy Limestone | – 8.4 | – 8.0 |
| Jhaba | J2 | Sandy Limestone | – 7.4 | – 9.1 |
|  |  | Sandy Limestone_Rep | – 7.5 | – 9.3 |
| Padlya | P7 | Eggshell | – 3.4 | – 12.5 |
|  |  | Sandy Limestone | – 8.7 | – 8.3 |
|  |  | Sandy Limestone_Rep | – 8.7 | – 8.4 |
|  | P16 | Sandy Limestone | – 6.4 | – 6.6 |
| Ottakoil, Cauvery Basin, Tamil Nadu, India | | | | |
| Ottakoil | Ot | Eggshell | – 2.6 | – 11.9 |
|  |  | Eggshell_Rep | – 2.7 | – 11.9 |

**S2 Supplementary data files**

Table S2.1 Diagnostic characters of the titanosaurid dinosaur clutches from the study areas of the Lameta Formation from Dhar (M.P.) District, India (abbreviations stand for A-Akhada, DR-Dholiya Raipuriya, J-Jhaba, JMP-Jamniapura, P-Padlya).

| **Nest#** | **Nest type/**  **material** | **No. of eggs** | **Min to max diameter of egg outline/egg (in cm)** | **Shape of egg outline/egg** | **Eggshells** | **Hatching windows** | **Shell pile/Double bottom** | **Egg type** |
| --- | --- | --- | --- | --- | --- | --- | --- | --- |
| A1 | Eggshells | 1 | - | - | Random | - | - | - |
| A2 | Eggshells | 1 | - | - | Random | - | - | - |
| A3 | Eggshells | 1 | - | - | Random | - | - | - |
| A4 | Eggshells | 1 | - | - | Random | - | - | - |
| A5 | Eggshells | 1 | - | - | Random | - | - | - |
| DR1 | Eggshells | 1 | - | - | Random | - | - | - |
| DR2 | Linear | 3 | 7-15 | Elliptical | Outside (concentric) and inside egg outline | - | Egg vi | Fragmented and compressed (vi) |
| DR3 | Eggshells | 1 | - | - | Closely spaced and concentric | - | - | - |
| DR4 | Circular | 3 | 6-10 | Elliptical | Random and inside | - | Egg i | Compressed (i); Bottom (ii, iv) |
| DR5 | Linear | 1 | - | - | Linear and concentric | - | - | - |
| DR6 | Linear | 2 | - | - | Linear | - | - | - |
| DR7 | Linear | 1 | - | - | Linear and concentric | - | - | - |
| DR8 | Linear | 4 | 5-17 | Elliptical | Linear and concentric | - | - | Compressed and fragmented (i) |
| DR9 | Combination | 5 | 6.1-15 | Sub-circular | Inside | Egg i | Egg v | Fragmented (i-v) |
| DR10 | Two eggs | 2 | 3.9-16.8 | Sub-circular to elliptical | Outside | Egg ii | - | Fragmented (i); Compressed (ii) |
| DR11 | Linear | 2 | - | - | Linear and concentric | - | - | - |
| DR12 | Eggshells | 1 | - | - | Random | - | - | - |
| DR13 | Two eggs | 2 | 3-15.8 | Sub-circular | Outside | - |  | Fragmented (i,ii) |
| DR14 | Eggshells | 1 | - | - | Random | - | - | - |
| DR15 | Linear | 1 | - | - | Linear and concentric | - | - | - |
| DR16 | Linear | 1 | - | - | Linear and concentric | - | - | - |
| J1 | Two eggs | 2 | 9.3-15.3 | Sub-circular | Outside | - | - | Partially intact and morphed (i,ii) |
| J2 | Two eggs | 2 | 5.5-14 | Elliptical | Inside and outside | - | Egg iii | Fragmented and compressed (i); Half-preserved (B) |
| J3 | Single egg | 1 | 16.2-16.5 | Circular | - | - | - | Fragmented |
| J4 | Circular | 4 | 10-14.8 | Sub-circular | Inside | - | - | Fragmented (i-iv) |
| J5 | Two eggs | 2 | 9.3-15.5 | Sub-circular | Inside and outside | - | - | Fragmented (i,ii) |
| J6 | Single egg | 1 | 13.2-13.5 | Circular | - | - | - | Partially intact and eroded |
| J7 | Circular | 4 | 11.3-15.3 | Elliptical | Inside | Egg i | Egg i | Compressed (i); Remnants (ii-iv) |
| J8 | Two eggs | 2 | 8.1-17 | Elliptical | Inside | Egg ii | Egg ii | Fragmented and compressed (i,ii) |
| J9 | Single egg | 1 | - | - | Outside | - | - | Bottom |
| J10 | Single egg | 1 | 11.1-12.7 | Sub-circular | Inside | Present | - | Fragmented |
| J11 | Circular | 3 | - | - | Inside | Eggs i,ii | Eggs i,ii | Half-preserved (i,ii); Compressed (ii); Remnant (iii) |
| J12 | Eggshells | 1 | - | - | Closely spaced | - | - | - |
| J13 | Single egg | 1 | 17.6-19 | Sub-circular | - | - | - | Unhatched |
| J14 | Two eggs | 2 | - | Sub-circular | - | - | - | Bottom (i,ii) |
| J15 | Single egg | 1 | 15-15.4 | Circular | - | - | - | Half-preserved |
| J16 | Single egg | 1 | - | - | - | - | - | Fragmented |
| J17 | Single egg | 1 | - | - | - | - | - | Stolen |
| J18 | Circular | 5 | - | - | - | - | - | Bottom; Other eggs stolen |
| JMP1 | Double eggs | 2 | 18 | Sub-circular | Outside | - | - | Fragmented |
| P1 | Combination | 20 | 9.8-18.8 | Sub-circular | - | Eggs ii,vi,vii,xiii-xvii | - | Intact (i,iii,viii,x,xi); Partially intact and half-preserved (ii,vi,vii,xiii); Bottom (iv,v,xii); Compressed (xi); Fragmented (xiv-xx) |
| P2 | Circular | 3 | 15.5-16.6 | Sub-circular | - | Egg i | - | Fragmented (i,ii); Remnant (iii) |
| P3 | Two eggs | 2 | 12.5-15.5 | Sub-circular | - | - | - | Fragmented (i,ii) |
| P4 | Single egg | 1 | - | - | - | - | - | Bottom |
| P5 | Single egg | 1 | 14.7-15.5 | Circular | - | - | - | Intact but eroded |
| P6 | Single egg | 1 | 16.1-16.8 | Sub-circular | - | - | - | Partially intact |
| P7 | Circular | 10 | 13-16.6 | Sub-circular to elliptical | Inside and outside | Eggs i,ii | Egg xiv | Half-preserved (i,ii); Unhatched (iii); Bottom (iv,vii); Intact and eroded (viii,ix); Remnant (L,M); Compressed (N) |
| P8 | Circular | 5 | 15-19 | Sub-circular | - | - | - | Partially intact (i,iii); Bowl-shaped (ii); Half-preserved (v); Bottom (iv) |
| P9 | Circular | 6 | 13.2-18.3 | Sub-circular to elliptical | - | - | - | Fragmented (i,ii,iv,v); Remnant (iii); Compressed (vi) |
| P10 | Two eggs | 2 | - | - | - | - | - | Bottom (i,ii) |
| P11 | Circular | 5 | 13.4-15.8 | Sub-circular | - | - | - | Bowl-shaped (i); Bottom (ii-v) |
| P12 | Circular | 4 | 13.1-19.6 | Sub-circular | - | - | - | Bottom (i,iv); Half-preserved (ii,iii) |
| P13 | Two eggs | 2 | 15-16.3 | Circular | Inside | - | - | Partially intact and eroded (i); Remnant (ii) |
| P14 | Combination | 4 | 12.7-18.3 | Sub-circular | Inside and outside | Eggs i-iv | Egg iv | Half-preserved and fragmented (i-iv) |
| P15 | Circular | 8 | 7.2-15.5 | Circular to sub-circular | Inside and outside | - | - | Partially intact (ii,iii,vii); Compressed (v); Bowl-shaped (vi); Half-preserved (viii) |
| P16 | Circular | 4 | 12-13.6 | Sub-circular | - | - | - | Fragmented (i); Bowl-shaped (iv) |
| P17 | Two eggs | 2 | 16.8-17.6 | Sub-circular | Inside | Egg ii | Egg ii | Fragmented and half-preserved (i,ii) |
| P18 | Single egg | 1 | 15.3-16 | Sub-circular | Outside | - | - | Partially intact and displaced |
| P19 | Circular | 3 | 10.5-13.5 | Sub-circular | Inside | Egg i | Egg i | Half-preserved (i,iii); Remnant (ii) |
| P20 | Circular | 8 | 9.5-14.5 | Sub-circular | Inside and outside | Eggs v,vi | Eggs v,vi | Unhatched (i,ii); Remnant (iii,vii); Fragmented (iv); Half-preserved (v,vi) |
| P21 | Circular | 4 | - | Sub-circular | - | - | - | Bottom (i-iv) |
| P22 | Circular | 14 | 4.5-15.8 | Sub-circular | Inside and outside | Eggs i,iv | Egg x | Half-preserved (i,iv,x); Bottom (ii); Fragmented (ix); Compressed (xi); Remnant (xii); Partially intact (xiv) |
| P23 | Two eggs | 2 | 8.2-13.5 | Sub-circular | Inside and outside | Egg iii | Egg iii | Fragmented (ii,iii) |
| P24 | Circular | 4 | 9-10 | Sub-circular | Outside | - | - | Fragmented (ii); Remnants (iii,iv) |
| P25 | Circular | 3 | 15-18.8 | Sub-circular to elliptical | Inside | Eggs i-iii | Eggs i,iii | Fragmented (i-iii) |
| P26 | Circular | 8 | 8.5-16.1 | Sub-circular to elliptical | Inside and outside | - | - | Fragmented (i,iii,x); Bottom (ii); Compressed (xi) |
| P27 | Eggshells | 1 | - | - | Random | - | - | - |
| P28 | Single egg | 1 | 13.1 | - | Inside | Present | Present | Half-preserved |
| P29 | Single egg | 1 | 17.7-19.3 | Sub-circular | - | - | - | Unhatched |
| P30 | Single egg | 1 | 16.7-20.3 | Elliptical | - | - | - | Unhatched and deformed |
| P31 | Single egg | 1 | 8.5-17.5 | Elliptical | Inside | Present | Present | Half-preserved |
| P32 | Single egg | 1 | 14.2-17.2 | Elliptical | - | - | - | Unhatched |
| P33 | Two eggs | 2 | 13.3-16.4 | Circular | - | - | - | Intact and eroded (i,ii) |
| P34 | Two eggs | 2 | 5.8-15 | Sub-circular | - | - | - | Bottom (i,ii) |
| P35 | Circular | 6 | 8.3-16.3 | Circular to sub-circular | - | - | - | Intact (i,ii,iii,v); Bottom (iv); Small sized (vi) |
| P36 | Eggshells | 1 | - | - | Random | - | - | - |
| P37 | Single egg | 1 | 16.3-18.6 | Sub-circular | - | - | - | Unhatched |
| P38 | Two eggs | 2 | 10.2 | - | Inside | Egg i | Egg i | Half-preserved (i,ii) |
| P39 | Single egg | 1 | - | - | - | - | - | Bottom |
| P40 | Two eggs | 2 | - | - | - | - | - | Bottom (i,ii) |
| P41 | Single egg | 1 | - | - | - | - | - | Bottom |
| P42 | Eggshells | 1 | - | - | Random | - | - | - |
| P43 | Single egg | 1 | 16.9-17 | Circular | - | - | - | Unhatched |
| P44 | Two eggs | 2 | 11.5-12.1 | - | - | - | - | Half-preserved (i,ii) |
| P45 | Eggshell | 1 | - | - | Single | - | - | - |
| P46 | Two eggs | 2 | 14.2 | - | - | - | - | Bowl-shaped (i) |
| P47 | Circular | 5 | - | - | - | - | - | Bottom (i-v) |
| P48 | Two eggs | 2 | - | - | Closely spaced | - | - | Bottom (i) |
| P49 | Two eggs | 2 | - | - | - | - | - | Bottom (i,ii) |
| P50 | Single egg | 1 | 14 | - | Inside | Present | Present | Half-preserved |
| P51 | Circular | 12 | 10.4-15.2 | Sub-circular to circular | Outside | - | - | Bottom (i-iv,ix,xii); Remnants (v-xi); Half-bowl (viii) |
| P52 | Circular | 3 | 9-13.5 | Sub-circular | - | - | - | Fragmented (i); Partially intact (ii,iii) |

Table S2.2 Eggshell dimensions of the oospecies recorded from the study areas.

| **Nest#** | **Thickness (in mm)** | **Height/Width (H/W)** | **Average node diameter (in mm)** | **Average basal cap diameter (in mm)** | **Oospecies** |
| --- | --- | --- | --- | --- | --- |
| A1 | 1.0-1.2 | 2.8:1 | 0.5 | 0.4 | *Fusioolithus baghensis* |
| A3 | 2.2-2.7 | 4.1:1 | 0.6 | 0.3 | *Megaloolithus cylindricus* |
| A4 | 1.4-1.7 | 2.4:1 | 0.6 | 0.4 | *Fusioolithus baghensis* |
| A5 | 1.8-2.5 | 2.7:1 | 0.7 | 0.5 | *Fusioolithus padiyalensis* |
| DR1 | 2-2.2 | 2.9:1 | 0.7 | 0.3 | *Megaloolithus jabalpurensis* |
| DR6 | 2-2.1 | 5.2:1 | 0.6 | 0.2 | *Megaloolithus cylindricus* |
| DR8 | 0.7-1.9 | 3.8:1 | 0.6 | 0.3 | *Megaloolithus jabalpurensis* |
| DR9 | 1.2-1.5 | 3.7:1 | 0.4 | 0.2 | *Fusioolithus mohabeyi* |
| DR10 | 1.7-2.1 | 3.5:1 | 0.6 | 0.4 | *Fusioolithus padiyalensis* |
| DR11 | 1.5-2 | 2.8:1 | 0.6 | 0.6 | *Megaloolithus jabalpurensis* |
| DR13 | 0.8-1.1 | 1.5:1 | 0.8 | 0.6 | *Megaloolithus cylindricus* |
| DR14 | 1.1-1.4 | 3.5:1 | 0.3 | 0.2 | *Megaloolithus cylindricus* |
| J9 | 1.1-1.8 | 3.6:1 | 0.4 | 0.3 | *Fusioolithus baghensis* |
| J10 | 0.8-1.2 | 2:1 | 0.5 | 0.2 | *Fusioolithus baghensis* |
| J11 | 0.4-1.1 | 2.7:1 | 0.5 | 0.2 | *Fusioolithus baghensis* |
| J12 | 2.1-2.3 | 2.8:1 | 1.0 | 0.2 | *Fusioolithus baghensis* |
| J13 | 2.1-3.2 | 4.5:1 | 0.7 | 0.3 | *Megaloolithus cylindricus* |
| J14 | 1.4-1.9 | 3.1:1 | 0.8 | 0.3 | *Megaloolithus dhoridungriensis* |
| J15 | 2.1-2.8 | 4.6:1 | 0.7 | 0.3 | *Fusioolithus padiyalensis* |
| J17 | 1-1.3 | 1.4:1 | 0.6 | 0.5 | *Megaloolithus jabalpurensis* |
| JMP1 | 1.4-2 | 1.4:1 | 0.9 | 0.3 | *Fusioolithus baghensis* |
| P3 | 1.5-1.7 | 3.4:1 | 0.9 | 0.4 | *Megaloolithus cylindricus* |
| P7 | 1.3-1.6 | 2.2:1 | 0.6 | 0.4 | *Megaloolithus jabalpurensis* |
| P8 | 0.9-1.5 | 3:1 | 0.6 | 0.2 | *Megaloolithus jabalpurensis* |
| P10 | 1.5-1.7 | 2.5:1 | 0.6 | 0.2 | *Megaloolithus jabalpurensis* |
| P11 | 1.4-1.6 | 2.6:1 | 0.6 | 0.4 | *Megaloolithus jabalpurensis* |
| P12 | 1.2-1.5 | 2.1:1 | 0.8 | 0.2 | *Megaloolithus dhoridungriensis* |
| P16 | 0.6-0.9 | 1.5:1 | 0.3 | 0.1 | *Megaloolithus jabalpurensis* |
| P19 | 1.6-1.8 | 2.6:1 | 0.6 | 0.4 | *Megaloolithus cylindricus* |
| P22 | 1.3-1.8 | 2:1 | 0.8 | 0.5 | *Fusioolithus mohabeyi* |
| P23 | 0.4-0.9 | 1.2:1 | 0.7 | 0.2 | *Fusioolithus baghensis* |
| P25 | 1.2-1.3 | 2.1:1 | 0.6 | 0.4 | *Fusioolithus baghensis* |
| P27 | 1-1.5 | 2.5:1 | 0.5 | 0.2 | *Fusioolithus mohabeyi* |
| P28 | 1.9-2 | 2.8:1 | 0.5 | 0.3 | *Megaloolithus cylindricus* |
| P29 | 1.6-2.1 | 5.2:1 | 0.4 | 0.2 | *Megaloolithus cylindricus* |
| P30 | 1.3-1.5 | 2.1:1 | 0.5 | 0.3 | *Megaloolithus cylindricus* |
| P32 | 1.8-2.1 | 4.2:1 | 0.6 | 0.3 | *Megaloolithus cylindricus* |
| P37 | 1.6-1.9 | 2.2:1 | 0.8 | 0.5 | *Megaloolithus jabalpurensis* |
| P39 | 0.7-1.5 | 2.1:1 | 0.7 | 0.3 | *Megaloolithus jabalpurensis* |
| P40 | 0.8-1.1 | 2.7:1 | 0.5 | 0.4 | *Fusioolithus baghensis* |
| P42 | 1.5-2.3 | 5.7:1 | 0.6 | 0.2 | *Megaloolithus cylindricus* |
| P44 | 0.9-1.8 | 3.6:1 | 0.6 | 0.3 | *Fusioolithus mohabeyi* |
| P47 | 1.9-2.2 | 3.6:1 | 0.7 | 0.2 | *Megaloolithus dhoridungriensis* |
| P49 | 1.6-1.8 | 2.5:1 | 0.5 | 0.3 | *Megaloolithus cylindricus* |
| P51 | 1.7-2.1 | 3.5:1 | 0.6 | 0.3 | *Megaloolithus cylindricus* |
| P52 | 1-1.2 | 2:1 | 0.6 | 0.1 | *Fusioolithus baghensis* |


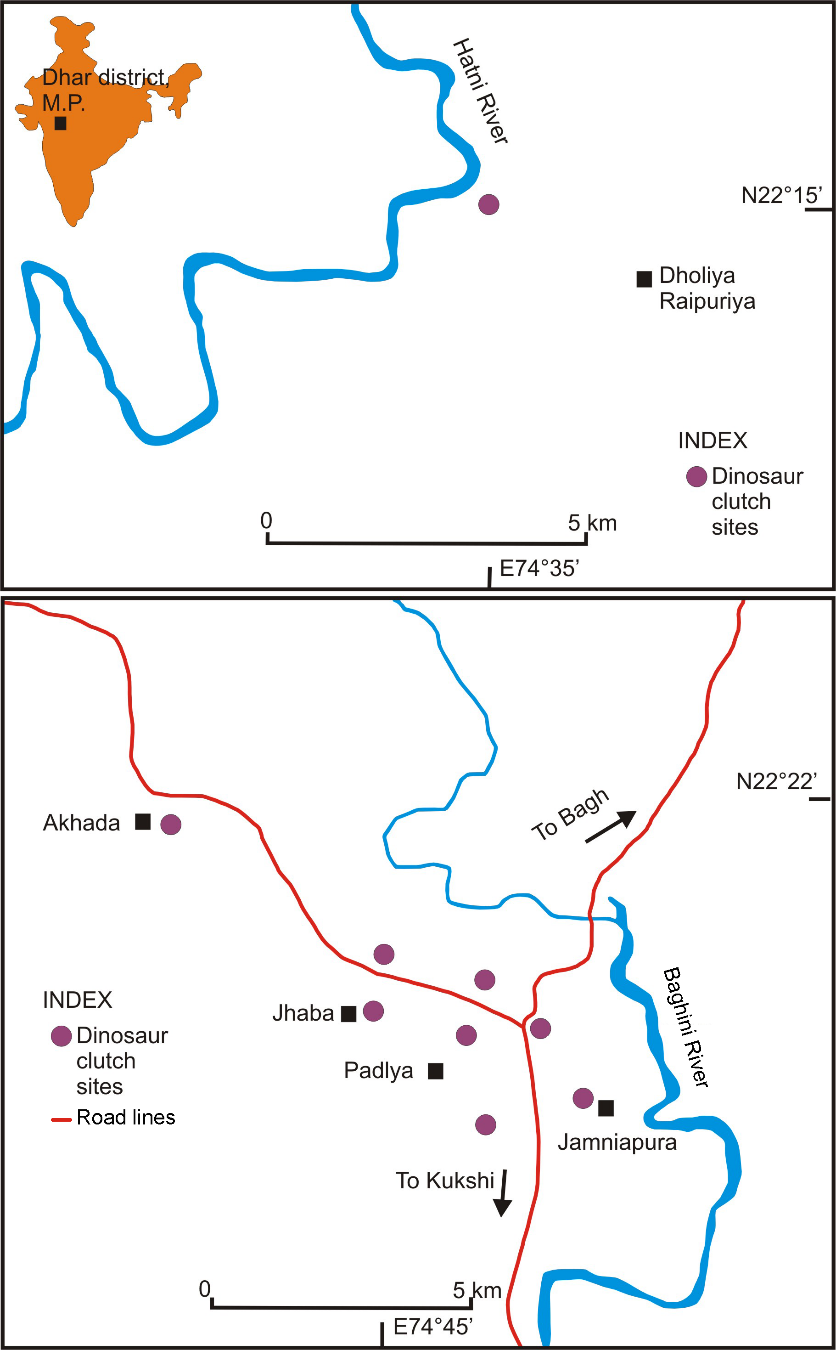


Figure S2.1 Map of the study area displaying the location of investigated dinosaur clutches (Courtesy of the University of Texas Libraries, The University of Texas at Austin).


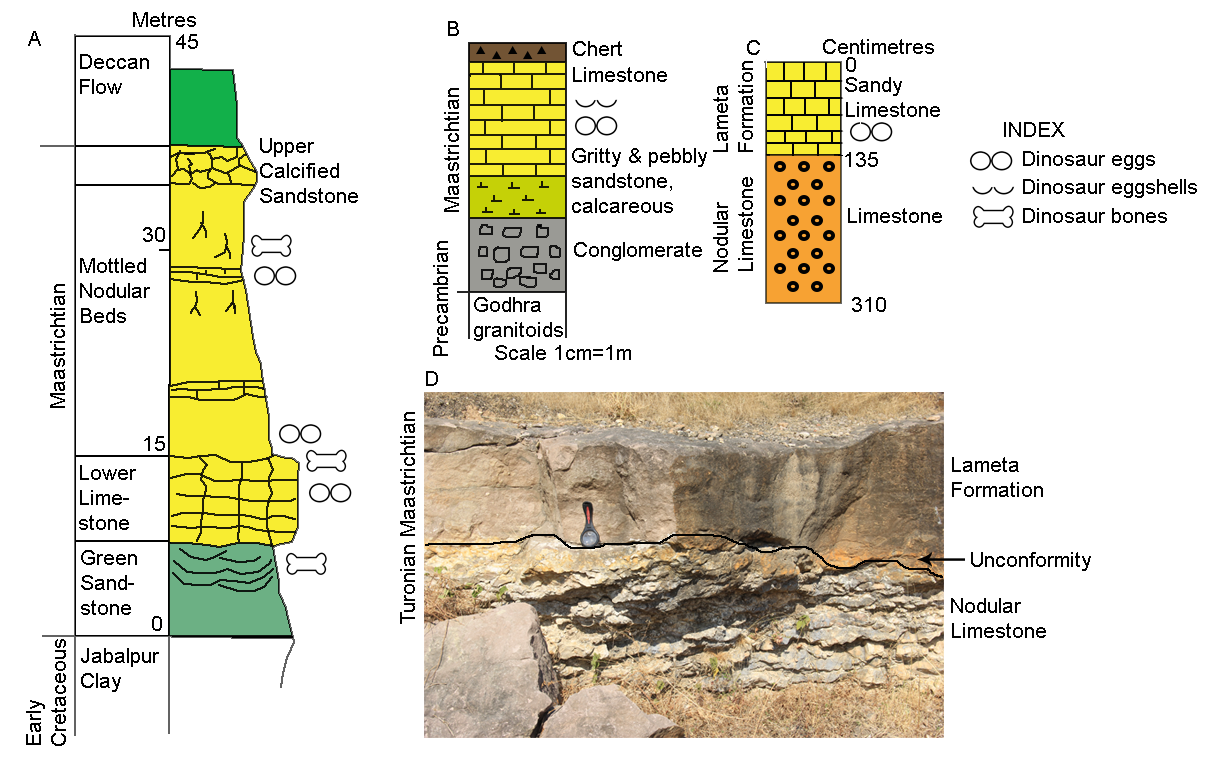


Figure S2.2 Stratigraphic subdivisions of the Lameta Formation in the type section at Jabalpur (A) (modified after Tandon et al. 1995), Rahioli (B) (modified after Srivastava et al. 1986), and Padlya, Dhar District, M.P. (C) (modified after Dhiman et al. 2022). (D) Field photograph showing the Sandy Limestone of the Lameta Formation overlying the Nodular Limestone of the Bagh Group within Dinosaur Fossil National Park (DFNP), near Padlya, Dhar District, M.P. The Nodular Limestone shows lensoid character while the sandy limestone has massive appearance.


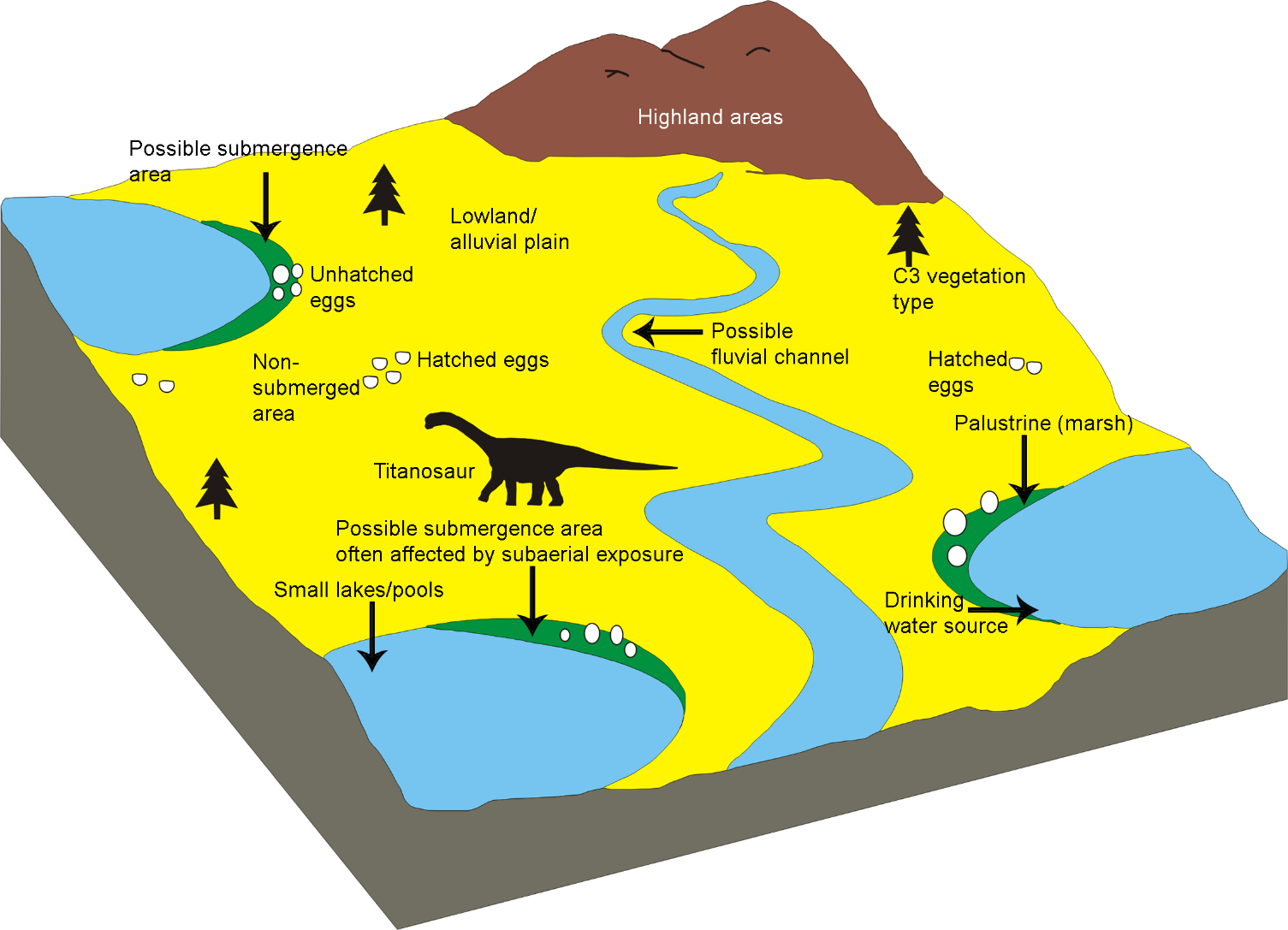


Figure S2.3 A block diagram showing the interpreted depositional environment of the Lameta Formation in the study areas. It is inferred that some of the clutches were laid close to the banks of the aquatic bodies (lakes/ponds) while others were deposited away from the lakes or ponds. The clutches laid close to the margins were prone to frequent submergence by water and thus got buried under sediment and remained unhatched, while the clutches laid away from the margins could hatch and hence showed more broken eggshells.

**References**

Basu S, Agrawal S, Sanyal P, Mahato P, Kumar S, Sarkar A. Carbon isotopic ratios of modern C3–C4 plants from the Gangetic Plain, India and its implications to paleovegetational reconstruction. Palaeogeography, Palaeoclimatology, Palaeoecology. 2015;440:22-32.

Brookfield ME, Sahni A. Palaeoenvironments of the Lameta Beds (Late Cretaceous) at Jabalpur, Madhya Pradesh, India: Soils and biotas of a semi-arid alluvial plain. Cretaceous Research. 1987;8(1):1-4.

Dhiman H, Verma V, Prasad GVR. First ovum-in-ovo pathological titanosaurid egg throws light on the reproductive biology of sauropod dinosaurs. Scientific Reports. 2022;12: 9362.

Dzombak RM, Sheldon ND, Mohabey DM, Samant B. Stable climate in India during Deccan volcanism suggests limited influence on K–Pg extinction. Gondwana Research. 2020;85:19-31.

Erben HK, Hoefs J, Wedepohl KH. Paleobiological and isotopic studies of eggshells from a declining dinosaur species. Paleobiology. 1979;5(4):380-414.

Jha DK, Sanyal P, Philippe A. Multi-proxy evidence of Late Quaternary climate and vegetational history of north-central India: Implication for the Paleolithic to Neolithic phases. Quaternary Science Reviews. 2020;229:106121.

Keith ML, Weber JN. Carbon and oxygen isotopic composition of selected limestones and fossils. Geochimica et cosmochimica acta. 1964;28(10-11):1787-816.

Kumari A, Singh S, Khosla A. Palaeosols and palaeoclimate reconstruction of the Maastrichtian Lameta Formation, Central India. Cretaceous Research. 2021;117:104632.

Montanari S, Higgins P, Norell MA. Dinosaur eggshell and tooth enamel geochemistry as an indicator of Mongolian Late Cretaceous paleoenvironments. Palaeogeography, Palaeoclimatology, Palaeoecology. 2013;370:158-66.

Platt NH, Wright VP. Palustrine carbonates and the Florida Everglades; towards an exposure index for the fresh-water environment?. Journal of Sedimentary Research. 1992;62(6):1058-71.

Riera V, Anadón P, Oms O, Estrada R, Maestro E. Dinosaur eggshell isotope geochemistry as tools of palaeoenvironmental reconstruction for the upper Cretaceous from the Tremp Formation (Southern Pyrenees). Sedimentary Geology. 2013;294:356-70.

Sarkar A, Bhattacharya SK, Mohabey DM. Stable-isotope analyses of dinosaur eggshells: Paleoenvironmental implications. Geology. 1991 Nov 1;19(11):1068-71.

Srivastava S, Mohabey DM, Sahni A, Pant SC. Upper Cretaceous Dinosaur egg clutches from Khede District (Gujarat, India), their distribution, shell ultrastructure and palaeoecology. Palaeontographica. Abteilung A, Paläozoologie, Stratigraphie. 1986;193(5-6): 219-33.

Tandon SK, Andrews JE. Lithofacies associations and stable isotopes of palustrine and calcrete carbonates: examples from an Indian Maastrichtian regolith. Sedimentology. 2001;48(2):339-55.

Tandon SK, Sood A, Andrews JE, Dennis PF. Palaeoenvironments of the dinosaur-bearing lameta beds (Maastrichtian), Narmada valley, central India. Palaeogeography, Palaeoclimatology, Palaeoecology. 1995;117(3-4):153-84.
